# Role of MRI in staging and follow-up of endometrial and cervical cancer: pitfalls and mimickers

**DOI:** 10.1186/s13244-019-0696-8

**Published:** 2019-02-13

**Authors:** María Milagros Otero-García, Alicia Mesa-Álvarez, Olivera Nikolic, Patricia Blanco-Lobato, Marijana Basta-Nikolic, Rafael Menéndez de Llano-Ortega, Laura Paredes-Velázquez, Nikola Nikolic, Magda Szewczyk-Bieda

**Affiliations:** 1 Department of Radiology, Hospital Universitario de Vigo , Carretera Clara Campoamor 341, 36312 Vigo, Spain; 2 Department of Radiology, Hospital Universitario Central de Asturias, Oviedo, Spain; 3 Clinical Centre of Vojvodina, Centre of Radiology, Faculty of Medicine, University of Novi Sad, Novi Sad, Serbia; 4 Centre of Radiology, Clinical Centre of Vojvodina, Novi Sad, Serbia; 5 Department of Clinical Radiology, Ninewells Hospital and Medical School, Dundee, UK

**Keywords:** Endometrial cancer, Cervical cancer, Magnetic resonance, Diffusion, Lymph nodes

## Abstract

MRI plays important roles in endometrial and cervical cancer assessment, from detection to recurrent disease evaluation. Endometrial cancer (EC) is the most common malignant tumor of the female genital tract in Western countries. EC patients are divided into risk categories based on histopathological tumor type, grade, and myometrial invasion depth. EC is surgically staged using the International Federation of Gynecology and Obstetrics (FIGO) system. Since FIGO (2009) stage correlates with prognosis, preoperative staging is essential for tailored treatment. MRI reveals myometrial invasion depth, which correlates with tumor grade and lymph node metastases, and thus correlates with prognosis. Cervical cancer (CC) is the second most common cancer, and the third leading cause of cancer-related death among females in developing countries. The FIGO Gynecologic Oncology Committee recently revised its CC staging guidelines, allowing staging based on imaging and pathological findings when available. The revised FIGO (2018) staging includes node involvement and thus enables both therapy selection and evaluation, prognosis estimation, and calculation of end results. MRI can accurately assess prognostic indicators, e.g., tumor size, parametrial invasion, pelvic sidewall, and lymph node invasion. Despite these important roles of MRI, radiologists still face challenges due to the technical and interpretation pitfalls of MRI during all phases of endometrial and cervical cancer evaluation. Awareness of mimics that can simulate both cancers is critical. With careful application, functional MRI with DWI and DCE sequences can help establish a correct diagnosis, although it is sometimes necessary to perform biopsy and histopathological analysis.

## Keypoints

- MRI of endometrial and cervical cancer facilitates patient stratification into treatment groups.
- MRI acquisition and interpretation errors can lead to diagnostic and staging mistakes.
- In endometrial and cervical cancer, DWI, and DCE improve staging accuracy and tumor delineation.
- For both endometrial and cervical cancer, assessing lymph node involvement plays an important role. Compared to CT and MRI, ^18f^luorine-18 fluorodeoxyglucose PET-CT (^18^F-FDG PET-CT) is more accurate for the detection of nodal metastasis larger than 10 mm.

## Introduction

At most institutions, the clinical use of MRI remains limited to specific clinical questions and selected patients. Notably, in cases of endometrial and cervical cancer, MRI offers the best diagnostic accuracy for staging and assessing lymphadenopathies, defining advanced disease, planning radiation ports, monitoring treatment response, and post-treatment surveillance to detect recurrence [1, 2]. In this context, MRI plays an important role in guiding primary treatment and reportedly improves patient outcomes [2–15].

Conventional MRI is useful for assessing the anatomical details of pelvic structures, but also has limitations and pitfalls [4, 7, 11, 16]. In the present review, we aimed to describe the important roles of MRI in endometrial and cervical cancer and to highlight the most common MRI pitfalls and mimickers, to help avoid mistakes that may impact patient management.

## Endometrial cancer

Endometrial cancer (EC) is the most common malignant tumor of the female genital tract in Western countries. Most cases are diagnosed at an early stage, and 75% occur in postmenopausal women (> 50 years) with vaginal bleeding being the main symptom [1, 4, 7, 8]. Risk factors include conditions promoting increased estrogen exposure, such as hormonal replacement therapy, obesity, tamoxifen use, early menarche, late menopause, nulliparity, history of polycystic ovary disease, and hereditary non-polyposis colorectal cancer (Lynch syndrome type 2) [3, 6, 17–19].

EC is categorized into two histopathological subtypes: type I (80–85%) and type II (10–15%). Type I is estrogen-dependent, affects younger patients (premenopausal or perimenopausal women), and is usually diagnosed at an early stage due to abnormal vaginal bleeding. Histologically, type I EC is a grade 1–2 endometrioid adenocarcinoma with a good prognosis (5-year survival, 80%). On the other hand, type II EC affects older women (postmenopause), is more commonly diagnosed at an advanced stage (60%) and can lead to the development of peritoneal carcinomatosis (as also occurs in ovarian cancer). Histologically, type II EC includes grade 3 endometrioid adenocarcinomas and other rare etiologies, such as clear cell carcinoma, undifferentiated serous carcinoma, and carcinosarcoma. Type II EC exhibits aggressive behavior and a poor prognosis (5-year survival, 40%) [4, 8, 17–19].

Recurrence is defined as tumor regrowth and/or the presence of distant disease after treatment. Early detection of recurrent disease is critical for establishing a therapeutic strategy with curative intent. Therefore, it is important to identify patients with a high risk of recurrence. Risk factors include advanced stage at diagnosis, high-grade disease, and lymphovascular space invasion (LVSI). Tumor recurrence typically occurs within 3 years after surgery (87%), with the most common recurrence sites being the vaginal vault (42%) and regional lymph nodes (LNs) (46%) [20].

## Cervical cancer

Cervical cancer (CC) is the second most commonly diagnosed cancer and the third leading cause of cancer-related deaths among females in developing countries. CC most often affects women between 45 and 55 years of age. Rates of CC show large geographic variation, reflecting differences in screening availability and in the prevalence of human papillomavirus (HPV) infection (particularly the oncogenic subtypes, e.g., HPV 16 and 18), which is detected in 99% of cervical tumors [10, 11, 17, 18, 21].

Squamous cell carcinoma comprises approximately 69% of all cervical cancers. The second most common type is adenocarcinoma, accounting for approximately 25% of CC. The remaining cases comprise rare histological types, including small cell neuroendocrine carcinoma and other epithelial tumors [17, 18]. The histological subtype and differentiation grade determine the disease course, the therapeutic outcome, and patient survival [17, 22, 23]. Though this remains controversial, the majority of studies show worse survival with adenocarcinomas compared to squamous cell carcinomas, with 10–20% differences in 5-year overall survival rates [24].

In cases of CC, 60–70% of post-treatment recurrences occur within 2 years post-treatment and 89–98% within 5 years after treatment. The four factors that most strongly predict recurrence are size greater than 3 cm, adenocarcinoma type, LVSI, and deep stromal invasion [10]. The most frequent recurrence sites are the pelvis (vaginal vault, cervix, parametrium, and pelvic wall) and the paraaortic LNs [25, 26].

## Role of MRI in endometrial and cervical cancers

### Detecting and staging endometrial cancer

In patients with abnormal vaginal bleeding, the endometrium is initially evaluated by transvaginal ultrasonography (TVUS). In postmenopausal patients with metrorrhagia, focal, or diffuse endometrial thickening of > 4 mm should be considered suspicious [27]. TVUS can be helpful in preoperative staging, showing an overall accuracy of 60–76% with regards to assessing the degree of myometrial invasion. It is also useful for evaluating cervical involvement, having a diagnostic accuracy comparable to contrast-enhanced MRI. Definitive EC diagnosis requires endometrial biopsy or dilation and curettage [3, 8, 27].

MRI is the best tool for preoperatively assessing myometrial invasion depth and cervical involvement—which correlate with tumor grade, presence of LN metastases, and overall survival [4, 8, 19]. The American College of Radiology recommends MRI as the preferred imaging modality for treatment planning, while the National Comprehensive Cancer Network (NCCN) guidelines advise MRI only in cases of type II endometrial cancer or suspected cervical invasion [28, 29]. The European Society of Urogenital Radiology (ESUR) recommends MRI in cases of type I endometrial carcinoma to identify patients with stage IA disease who would not benefit from lymphadenectomy, in cases of type II carcinomas to detect extrauterine spread, and in patients of childbearing age with grade 1 endometrioid adenocarcinoma to identify those with endometrium-confined disease who could benefit from fertility-sparing treatment [8].

EC is surgically staged using the International Federation of Gynecology and Obstetrics (FIGO) (2009) and TNM (8th Edition) systems (Table 1) [30, 31]. Although FIGO stage correlates with prognosis, preoperative staging is essential for tailored treatment. The standard surgical staging procedure includes hysterectomy, bilateral salpingo-oophorectomy, LN dissection, peritoneal washing, and omental biopsies. Patients with an intermediate or high risk of LN metastasis (type I EC with myometrial invasion of ≥ 50% or type II EC) benefit from lymphadenectomy [4–6, 8]. The European Society of Medical Oncology (ESMO) guidelines do not recommend lymphadenectomy in low-risk patients (type I EC with myometrial invasion of < 50%).

**Table 1:** TNM (8th Edition) and FIGO (2009) Classification of endometrial cancer (from refs. [30, 31])

TNM	FIGO	Description
Tx		Primary tumor cannot be assessed
T0		No evidence of primary tumor
T1	I^a^	Tumor confined to the corpus uteri^a^
T1a	IA^a^	Tumor limited to the endometrium or invading less than half of the myometrium
T1b	IB	Tumor invades one half or more of the myometrium
T2	II	Tumor invades cervical stroma, but does not extend beyond the uterus
T3	III	Local and/or regional spread
T3a	IIIa	Tumor invades the serosa of the corpus uteri or adnexa (direct extension or metastasis)
T3b	IIIb	Vaginal or parametrial involvement (direct extension or metastasis)
N1, N2	IIIC	Metastasis to pelvic or paraaortic lymph nodes^b^
N1	IIIC1	Metastasis to pelvic lymph nodes
N2	IIIC2	Metastasis to paraaortic lymph nodes with/without metastasis to pelvic lymph nodes
T4^c^	IVA	Tumor invades bladder/bowel mucosa
M1	IVB	Distant metastasis (excluding metastasis to vagina, pelvic serosa, or adnexa) (including metastasis to inguinal lymph nodes, intra-abdominal lymph nodes other than paraaortic or pelvic nodes)

^a^Endocervical glandular involvement alone should be considered stage I

^b^Positive cytology must be reported separately without affecting the stage

^c^The presence of bullous edema is not sufficient evidence to classify as T4

Computed tomography (CT) can detect pathological LNs and metastatic disease beyond the pelvis. Fluorine-18 fluorodeoxyglucose (^18^F-FDG) PET-CT is useful for detecting distant metastatic deposits (sensitivity of 100% and specificity of 94%) and assessing nodal disease [32].

### Detecting and staging cervical cancer

Until 2018, CC was clinically staged based on the FIGO 2009 classification. However, in 2018, the FIGO Gynecologic Oncology Committee made revisions to allow stage assignment based on imaging and pathological findings, when available [26]. Table 2 shows the revised staging (FIGO 2018) and TNM (8th Edition) classifications [26, 31].

**Table 2:** TNM (8^th^ Edition) and FIGO (2018) Classification of cervical cancer (from refs. 26, 31)

TNM	FIGO	Description
Tx		Primary tumor cannot be assessed
T0		No evidence of primary tumor
Tis		Preinvasive carcinoma
T1	I	The carcinoma is strictly confined to the cervix (extension to the uterine corpus should be disregarded)
T1a	IA	Invasive carcinoma that can be diagnosed only by microscopy, with maximum depth of invasion < 5 mm^a^
T1a1	IA1	Measured stromal invasion depth of < 3 mm
T1a2	IA2	Measured stromal invasion depth ≥ 3 mm and < 5 mm
T1b	IB	Invasive carcinoma with measured deepest invasion of ≥ 5 mm (greater than Stage IA), lesion limited to the cervix uteri^b^
T1b1	IB1	Invasive carcinoma with measured deepest stromal invasion of ≥ 5 mm, and greatest dimension of < 2 cm
T1b2	IB2	Invasive carcinoma with greatest dimension of ≥ 2 cm and < 4 cm
-	IB3^d^	Invasive carcinoma with greatest dimension of > 4 cm
T2	II	The carcinoma invades beyond the uterus, but has not extended into the lower third of the vagina or to the pelvic wall
T2a	IIA	Involvement limited to the upper two-thirds of the vagina without parametrial invasion
T2a1	IIA1	Invasive carcinoma with greatest dimension of < 4 cm
T2a2	IIA2	Invasive carcinoma with greatest dimension of ≥ 4 cm
T2b	IIB	With parametrial involvement but not up to the pelvic wall
T3	III	The carcinoma involves the lower third of the vagina and/or extends to the pelvic wall and/or causes hydronephrosis or nonfunctioning kidney and/or involves pelvic and/or para-aortic lymph nodes^c^
T3a	IIIA	The carcinoma involves the lower third of the vagina, with no extension to the pelvic wall
T3b	IIIB	Extension to the pelvic wall and/or hydronephrosis or nonfunctioning kidney (unless known to be due to another cause)
N^d^	IIIC^d^	Involvement of pelvic and/or para-aortic lymph nodes, irrespective of tumor size and extent (with r and p notations)^c^
	IIIC1^d^	Pelvic lymph node metastasis only
	IIIC2^d^	Para-aortic lymph nodes metastasis
T4	IV	The carcinoma has extended beyond the true pelvis or has involved (biopsy proven) the mucosa of the bladder or rectum (the presence of bullous edema is not sufficient to classify a case as Stage IV)
	IVA	Spread to adjacent pelvic organs
M1	IVB	Spread to distant organs

When in doubt, the lower staging should be assigned

^a^Imaging and pathology can be used, when available, to supplement clinical findings with respect to tumor size and extent in all stages

^b^The involvement of vascular/lymphatic spaces does not change the staging. The lateral extent of the lesion is no longer considered

^c^The notations of r (imaging) and p (pathology) are added to indicate the findings used to assign a case as Stage IIIC. For example, if imaging indicates pelvic lymph node metastasis, the stage allocation would be Stage IIIC1r, whereas if confirmed by pathologic findings, the stage would be Stage IIIC1p. The type of imaging modality or pathology technique used should always be documented

^d^The revised FIGO classification was recently published (October 2018). TNM (8^th^ Edition) does not include classification for the new FIGO groups IB3, IIIC1, and IIIC2. TNM defines only regional lymph nodes, with N0 (i+) indicating isolated tumor cells in regional lymph node(s) no greater than 0.2 mm, and N1 indicating regional lymph node metastasis

Stage IA1 and IA2 cancers are diagnosed by microscopic examination of specimens from a loop electrosurgical excision procedure or cone biopsy. They can also be diagnosed from trachelectomy or hysterectomy specimens. Clinically visible lesions (stage 1B and higher) are diagnosed by punch biopsy. Some cases may require performance of a small loop biopsy or cone biopsy [26]. The revised FIGO staging also permits the use of available imaging modalities (ultrasound, CT, and MRI) to obtain information regarding tumor size, nodal status, and local or systemic spread [26].

In experienced hands, TVUS is highly accurate for evaluating cervical stroma infiltration in patients with early-stage CC. TVUS may also identify complications of local-regional invasion, such as hydronephrosis or endometrial cavity distension secondary to cervical canal obstruction by the tumor. On the other hand, the limited soft-tissue contrast and the small field of view (FOV) may lead to suboptimal evaluation of parametrial invasion, and therefore, TVUS plays a limited role in staging patients with CC [3].

MRI is the best method for assessing primary tumors over 10 mm in size, since it can accurately determine tumor size, parametrial invasion, pelvic sidewall invasion, and LN metastasis, with up to 95% accuracy for stage IB or higher [2, 10, 11, 26, 33]. In young patients who desire fertility preservation, MRI is necessary for evaluating the potential for conservative procedures. Eligibility criteria for vaginal radical trachelectomy (VRT) and abdominal radical trachelectomy (ART) include tumor size (≤ 2 cm for VRT, ≤ 4 cm for ART), and tumor distance from the internal cervical os (> 1 cm for VRT, > 0.5 cm for ART) [34]. Young women with larger tumors (FIGO IB1, IB2) are usually selected for ART or neoadjuvant chemotherapy plus conservative surgery [10, 35]. ART can also be performed in patients with limited vaginal access and in selected patients with early-stage cervical cancer at 15–17 weeks of gestation [36].

Compared to CT and MRI, ^18^F-FDG PET-CT is more accurate for the detection of nodal metastasis larger than 10 mm, with false-negative results in 4–15% of cases. To avoid false-positive results (especially in areas with high prevalences of tuberculosis and inflammation, particularly HIV-endemic areas), metastases can be established or excluded by fine-needle aspiration or biopsy of large LNs [26].

The risk of pelvic LN involvement increases with greater tumor size, from 6% for tumors with a maximal diameter of < 2 cm to 36% for tumors with a maximal diameter of > 4 cm [37]. In early-stage disease, ^18^F-FDG PET-CT has a sensitivity of 53–73% and specificity of 90–97% for detecting LN involvement. In more advanced stages, the sensitivity for detecting paraaortic node involvement increases to 75%, with 95% specificity [24]. Moreover, ^18^F-FDG PET-CT can identify local and distant metastasis and ensure that the radiation treatment volume encompasses both clinically involved and high-risk areas [10, 11, 38, 39].

Recent evidence suggests that sentinel LN biopsy (SLNB), with or without dissection, also has an important role in CC investigation [24, 39]. According to the revised FIGO, SLNB is still experimental and more evidence is needed to support its inclusion in routine practice. SLNB may be particularly useful in early-stage cervical cancer (FIGO stage IA, IB1, and IB2). Dual labeling using blue dye and radiocolloid increases the accuracy of sentinel LN detection, and a near-infrared technique using indocyanine green dye has been applied in robotic surgery and laparoscopy. Pelvic lymphadenectomy must be considered when LVSI is present [26].

ESUR and NCCN guidelines recommend the use of cross-sectional imaging techniques for staging CC in stage IB1 or greater [11, 29]. Moreover, the guidelines of the ESMO and the European Society of Gynaecological Oncology, in conjunction with the European Society for Radiotherapy and Oncology and the European Society of Pathology, recommend SLNB biopsy and/or dissection as appropriate [24, 39].

For patients with CC, treatment decisions are made based on the disease stage and extent of spread. Cervical cancer is primarily managed by surgery (abdominal and/or vaginal; laparoscopic or robotic), radiation therapy, and chemotherapy as adjunct therapy in advanced stages [26].

#### Microinvasive cervical cancer (FIGO IA)

In cases of stage IA1 without LVSI, the appropriate treatment is conization or VRT. In patients not wishing to preserve fertility, simple extrafascial hysterectomy is performed. To treat stage IA1 with LVSI, pelvic lymphadenectomy should be considered, along with modified radical hysterectomy [26].

In cases of stage IA2 tumors, fertility-sparing procedures include cervical conization with laparoscopic (or extraperitoneal) pelvic lymphadenectomy, ART, VRT, or laparoscopic trachelectomy with pelvic lymphadenectomy. For women not wishing to preserve fertility, pelvic lymphadenectomy is performed along with modified radical hysterectomy or more radical surgery. In low-risk cases, adequate surgical treatment may comprise simple hysterectomy or trachelectomy, with either pelvic lymphadenectomy or SLNB [26].

#### Invasive cervical carcinoma (FIGO stages IB1, IB2, and IIA1)

Women with tumors of ≤ 4 cm are usually treated with radical hysterectomy and systematic pelvic lymphadenectomy. For young women desiring fertility preservation and in FIGO stages IA2–IB1 (tumors ≤ 2 cm), radical trachelectomy (VRT, ART, or minimally invasive) may be performed. When a vaginal approach is planned, first, the pelvic nodes are laparoscopically removed and sent for frozen section analysis to confirm node negativity before proceeding with the radical trachelectomy. Alternatively, the nodes may be assessed using conventional pathologic methods, followed by the radical trachelectomy as a second surgery 1 week later [26].

#### Locally advanced CC (FIGO stages IB3, IIA2, IIB, III, and IVA)

For locally advanced CC, the preferred treatment option is concurrent chemoradiation (CCRT), which includes external radiation and intracavitary brachytherapy. In patients with only central disease, without pelvic sidewall involvement or distant metastasis (stage IVA disease and pelvic recurrence), pelvic exenteration can be considered but is usually associated with a poor prognosis [26].

#### FIGO stage IVB/distant metastasis

CC with distant metastatic disease occurs in about 2% of cases, and the median survival duration in such cases is approximately 7 months. In patients with positive paraaortic and supraclavicular nodes, CCRT reportedly elicits a better response than systemic chemotherapy (69% and 57%, respectively), and thus, CCRT is the elective treatment [26].

## MRI technique for endometrial and cervical cancers

The diagnostic performance of MRI, in terms of obtaining optimal imaging and avoiding pitfalls, depends on the use of appropriate sequences, using the correct imaging planes, and obtaining the highest contrast and spatial resolution. Tables 3 and 4 list the details of basic MRI protocols and most technical pitfalls.

**Table 3:** MRI protocol for endometrial and cervical carcinoma

Sequence	Axial T1	Sagittal T2	Sagittal DWI	Coronal T2	Axial T2^$^	Axial DWI	Axial DCE	Sagittal DCE	Axial T2	Coronal T2
Sequence	TSE	TSE	EPI	TSE	TSE	EPI	FS 3D GRE T1^%^	3D FS GRE T1	SS-FSE^&^	SS-FSE^&^
TE (ms)	8	100	90	100	100	81	2.3	2.3	70	70
TR (ms)	550	4000	4000	4000	4000	6000	4.5	3.6	1000	1000
Echo train length	4	17	EPI	17	17	EPI	–	–	55	55
Flip angle (degrees)	90	90	90	90	90	90	10	10	90	90
FOV (mm)	320	200	320	200	200	200	300	300	350	320
Slice Thickness (mm)	5	3	3	3	3	3	1.75	1.4	5	5
Gap (mm)	0.5	0.3	0.3	0.3	0.3	0.3	–	–	0.5	0.5
NEX	1	2	2	2	2	1/2/8/12	1	2	1	1
Matrix size	0.8 × 1	0.6 × 0.7	3 × 3	0.6 × 0.7	0.6 × 0.7	2.6 × 3	1.9 × 1.9	1.5 × 1.8	1.4 × 1.6	1.4 × 1.6
*b* values (s/mm^2^)			0, 800			0, 500, 800, 1000				

*DWI* diffusion-weighted imaging, *DCE* dynamic contrast-enhanced sequence, *TE* echo time, *TR* recovery time, *FOV* field of view, *NEX* number of excitations, *TSE* turbo spin echo, *EPI* echo planar, *3D FS GRE T1* 3D fat-saturated gradient-echo T1 sequence

We use the same protocol with different angulation in the axial oblique sequence (perpendicular to the endometrial cavity or cervix) or double oblique sequence (angled in both the sagittal and coronal planes)

^%^40 dynamics (8 s)

^$^If the uterus is tilted, we perform double axial plane imaging using sagittal and coronal T2WI sequences

^&^Single-shot fast-spin echo

**Table 4:** MRI technical artifacts. Pitfalls and pearls

Artifacts	Pitfall	Pearl
Motion artifacts Peristalsis (bladder, bowel)	Limit visualization of the anatomical detail of the uterus	• Adequate patient preparation • Use antiperistalsis agents
Sequence-specific artifacts while using rapid parallel imaging techniques such as ASSET (GE units) and SENSE (Phillips units), mSENSE (Siemens units)	May make image interpretation difficult	• Increase FOV
Metal artifacts - Hip prosthesis - Surgical clips	Limit visualization of the anatomical detail of the uterus and pelvic structures Makes staging more difficult	• Use MARS sequences

*FOV* field of view, *MARS* metal artifact reduction sequences

### Patient preparation

**Fig. 1:**
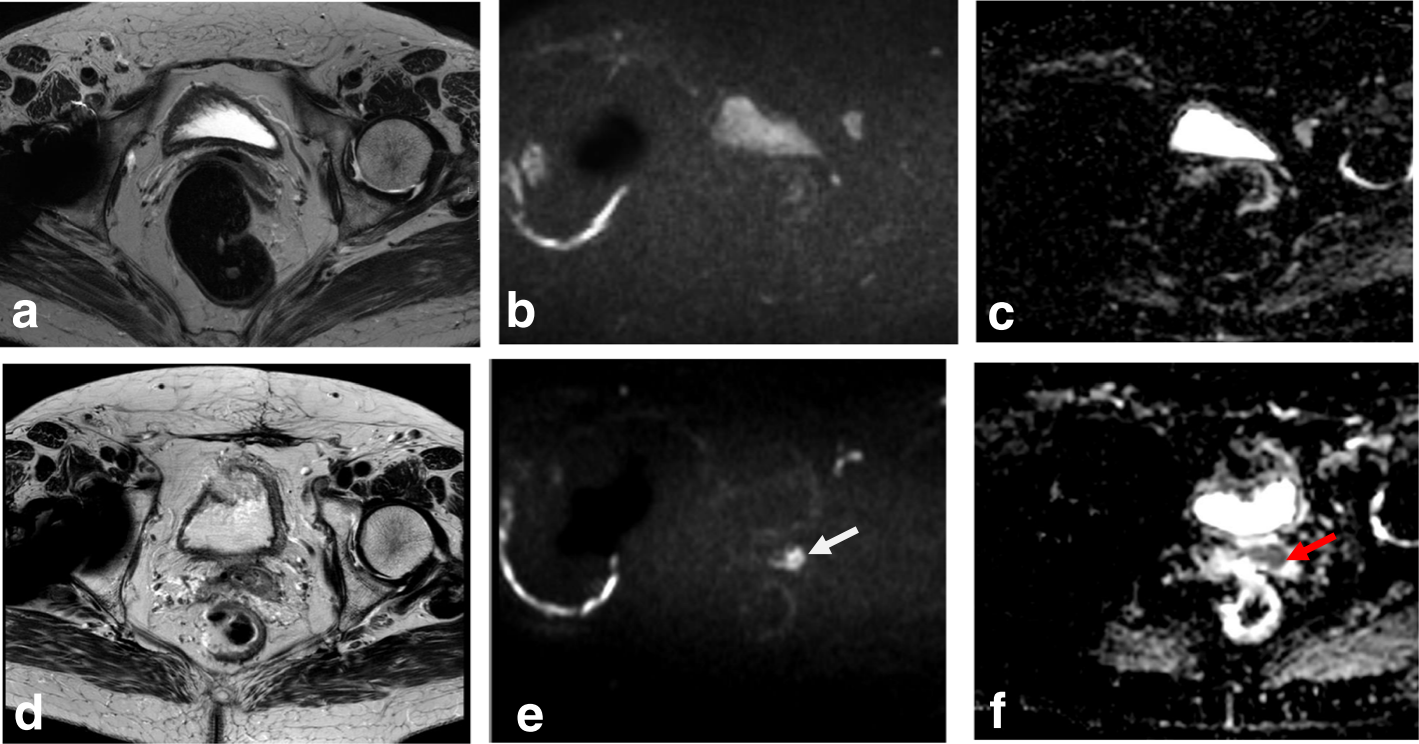
MRI technique artifacts. **a** Axial oblique T2WI. **b**, **c** DWI (b: 1000 and ADC map). Right hip prostheses and rectal air prevent detection of a small cervical tumor. **d**–**f** Axial oblique T2WI and DWI. After rectal air removal and change of phase direction, a tiny cervical cancer tumor is visible (arrows)

Prior to examination, it is recommended that patients be given a questionnaire or asked clinical questions regarding clinical symptoms, time of last menstruation, hormonal medication, and prior surgical procedures [40]. To improve MRI performance, patients must fast for 4–6 h and bladder and rectal voiding is advised to reduce motion artifacts (Fig. 1; Table 4). Motion artifacts from bowel and uterine peristalsis can be further reduced by intramuscular or intravenous injection of an antiperistalsis agent (hyoscine butylbromide or glucagon).

Patients should be imaged in the supine position, using 1.5 or 3.0 T MRI equipment with a body, pelvic, or cardiac phase-array surface coil. Fat saturation bands should be applied to eliminate motion artifacts from the anterior abdominal wall [2, 4, 8, 10, 16, 19]. Endorectal or endovaginal coils can provide high-resolution images of small cervical tumors, but their small FOV limits the assessment of large tumors, extrauterine extension, and pelvic LNs [41]. Vaginal opacification with gel is an optional measure that may be useful in cases with suspected cervical tumor extension into the vagina, particularly into the posterior vaginal fornix [42, 43].

### T2-weighted imaging

**Fig. 2:**
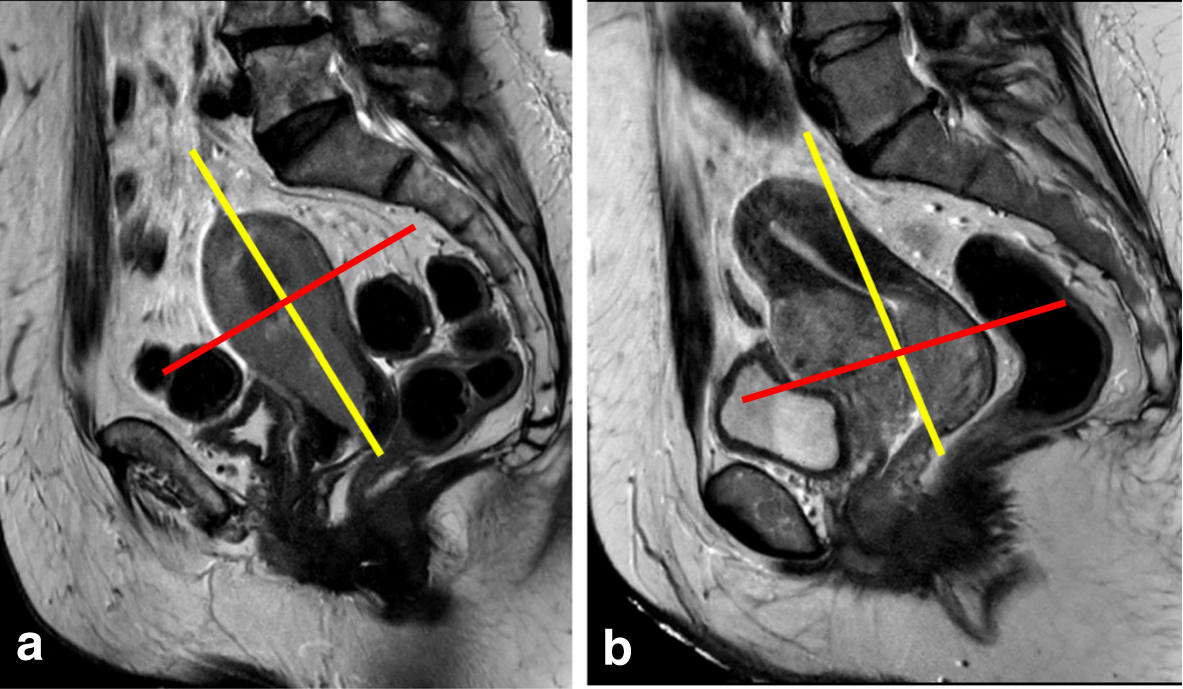
Planning of MRI sequences in endometrial (**a**) and cervical (**b**) cancers. Yellow line indicates the coronal plane, red line indicates the axial oblique plane

T2-weighted imaging (T2WI) is the mainstay of pelvic MRI. They are best performed without fat suppression (FS) due to the inherent contrast between the signal intensity (SI) of the uterus and the surrounding fat. Thin sections (3–4 mm) and a FOV of 20–24 cm are recommended. For T2WI, image acquisition must be optimized and angled perpendicularly to the endometrium or cervix (Fig. 2). To obtain axial oblique images of a tilted uterus, “double oblique images” angled in both the sagittal and coronal planes create a “true oblique” that is exactly orthogonal to the endometrial or endocervical cavities [8]. Axial/coronal T2WI or T1-weighted imaging (T1WI) from the renal hila to the pubic bone (36–44 cm) can be useful for assessing paraaortic lymphadenopathies, hydronephrosis, and bone metastases [4, 10, 11, 19].

### Functional imaging

Diffusion-weighted imaging (DWI) is necessary because it improves uterine tumor detection and characterization and the visualization of small implants in peritoneal carcinomatosis. The DWI protocol should include at least one plane, but preferably two planes (axial oblique along the uterus with the same orientation as axial oblique T2WI, and sagittal), with a minimum of two *b* values (e.g., *b* = 0, *b* = 1000). Acquiring T2WI and DWI on the same plane allows image fusion and optimizes anatomic correlation. To avoid pitfalls, the images from DWI should always be evaluated together with the corresponding ADC maps and anatomic images [4, 8, 10, 44].

Dynamic contrast-enhanced (DCE) images are obtained using a 3D FS gradient-echo (GRE) T1WI sequence, following intravenous administration of 0.1 mmol/kg gadolinium at a rate of 2–3 mL/s. Images are traditionally acquired in the axial oblique plane perpendicular to the body of the uterus (EC) or the sagittal plane (CC). Scanning is performed before contrast injection and then during multiple phases of enhancement, at 30, 60, 90, 120, and 150 s after the injection. For the detection of cervical stroma invasion while staging an EC, it is optimal to perform a delayed sequence acquired on the axial oblique plane, around 4 min after injection [2, 4, 8, 11, 19].

### Functional imaging in disease prognosis and treatment response monitoring

DWI and DCE sequences reflect changes in the oxygenation, perfusion, and tissue physiology of the tumor microstructure and yield quantitative and semi-quantitative parameters that can potentially serve as biomarkers of tumor characteristics. The ADC value and DCE have been investigated with regards to carcinoma aggressiveness and are reportedly related to prognosis. In theory, ADC values are lower in high-grade tumors due to their increased cellularity and restricted water diffusion [44–50].

In EC, Rechichi et al. [47] used two *b* values (*b* = 0, *b* = 1000) and an ADC value threshold of 1.05 × 10^3^ mm^2^/s to distinguish endometrial cancer from normal endometrial tissue. However, they found no correlation between ADC values and tumor grade. Bonatti et al. [51] recently demonstrated that the presence of deep myometrial infiltration and a tumor/uterus volume ratio of > 0.13 correlated with high-grade EC, whereas ADC values were not useful for predicting the histological grade of EC. Inada et al. [48] found that using DWI and T2WI improved the staging accuracy of myometrial invasion, with a sensitivity of up to 96%. In a recent meta-analysis of 15 studies including 849 patients, T2WI plus DWI showed superior specificity (0.947) compared to DCE imaging (0.86; *p =* 0.0035) [52].

Among CC patients, Somoye et al. [50] demonstrated that median mid-treatment ADC values were higher in survivors (1.55 × 10^−3^ mm^2^/s) than in non-survivors (1.36 × 10^−3^ mm^2^/s), with a 14% difference. Additionally, ADC values after 4 weeks of treatment reportedly correlate with volume and clinical response [53, 54]. Patients treated with neoadjuvant chemotherapy show early increases in ADC values, which negatively correlate with the proliferating cell nuclear antigen and cell density in patients who respond, indicating that ADC values are related to cellular features of the response that precedes size reduction [54].

Several studies show that high peak enhancement in pretreatment DCE is associated with tumor regression and local tumor control. High perfusion before and during radiotherapy suggests increased vascularity and high tumor oxygenation, which are both associated with better treatment response and prognosis [55–58]. Mayr et al. [59] demonstrated increased tumor perfusion or relative SI during early radiation therapy for cervical cancer, suggesting improved oxygenation of previously hypoxic cells, which would make radiotherapy more effective in these tumors. Upon therapy completion, persistent enhancement at the original cervical tumor site or in the post-surgical bed likely indicates residual disease, which is associated with increased risk of recurrence and poor survival [60].

#### Functional imaging in follow-up and detection of recurrent disease

The use of MRI with DCE imaging and DWI aids in recurrent tumor detection and allows differentiation from postradiotherapy changes [20, 45]. ^18^FDG PET-CT remains the imaging modality of choice for evaluating LNs and distant disease, having a sensitivity of 96% and specificity of 95% [61].

## MRI pitfalls in endometrial cancer

There are several commonly recognized signal characteristics of EC on MRI. In T2WI, an EC tumor appears as a diffuse or well-delineated soft tissue mass within the endometrial cavity, which shows heterogeneous intermediate SI relative to the hyperintense normal endometrium and hypointense myometrium. In DWI, tumors appear hyperintense at the high *b* value, with a corresponding hypointense signal on the ADC map. On DCE images, small tumors may show early enhancement compared to the normal endometrium, and slower enhancement than the myometrium. During later phases, these tumors may appear hypointense relative to the myometrium. Using DCE imaging, the presence of uninterrupted enhancement of the subendometrial zone is best evaluated at approximately 25–60 s after contrast injection. Myometrial invasion is best assessed during the equilibrium phase, at 2.5 min after contrast injection. An imaging delay of approximately 90 s is the optimal timing for assessing tumor-myometrium contrast. Delayed-phase images obtained around 4 min after contrast injection are useful for detecting cervical stromal invasion [4, 8, 19, 62, 63].

### Staging pitfalls

#### Detection and myometrial invasion (stage IA/IB)

**Fig. 3:**
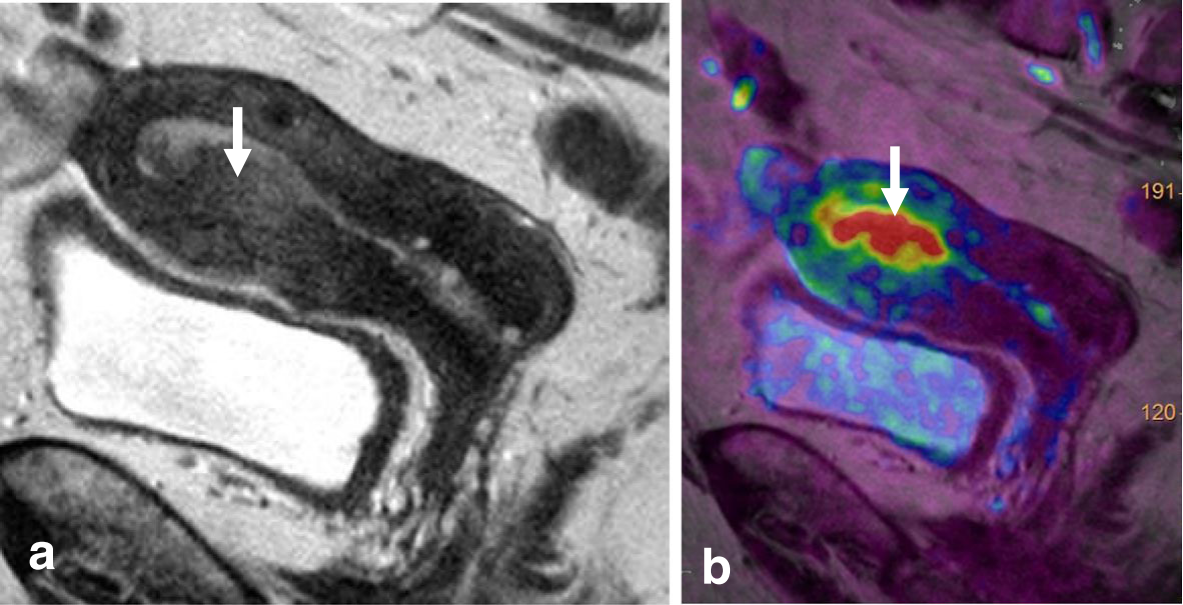
FIGO IA endometrial tumor (arrows) with myometrial invasion of < 50% is isointense in T2WI images (**a**) and well-delineated in fused T2WI-DWI images (**b**)

Local staging of EC requires evaluating the depth of tumor extension into the myometrium. Myometrial invasion is almost completely excluded by observation of an intact low-SI junctional zone on T2WI, and a smooth uninterrupted band of early subendometrial enhancement on DCE images [4, 8]. On the other hand, disruption of this subendometrial band indicates myometrial invasion. Invasion of < 50% of the myometrial thickness indicates a stage IA tumor, while invasion of ≥ 50% of the myometrial thickness indicates a stage IB tumor (Fig. 3) [4, 8, 19].

**Fig. 4:**
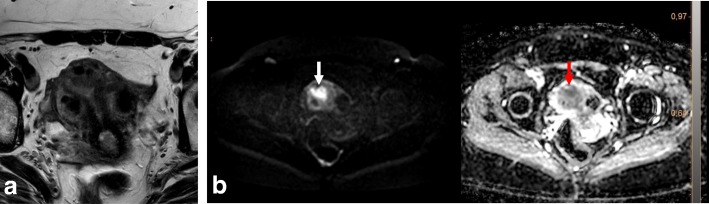
Uterus of postmenopausal woman exhibits myomas and FIGO IA endometrial cancer. Endometrial tumor is not delineated on axial oblique T2WI images (**a**) and is well-defined on DWI (*b* = 1000, ADC map) (arrows) (**b**)

**Fig. 5:**
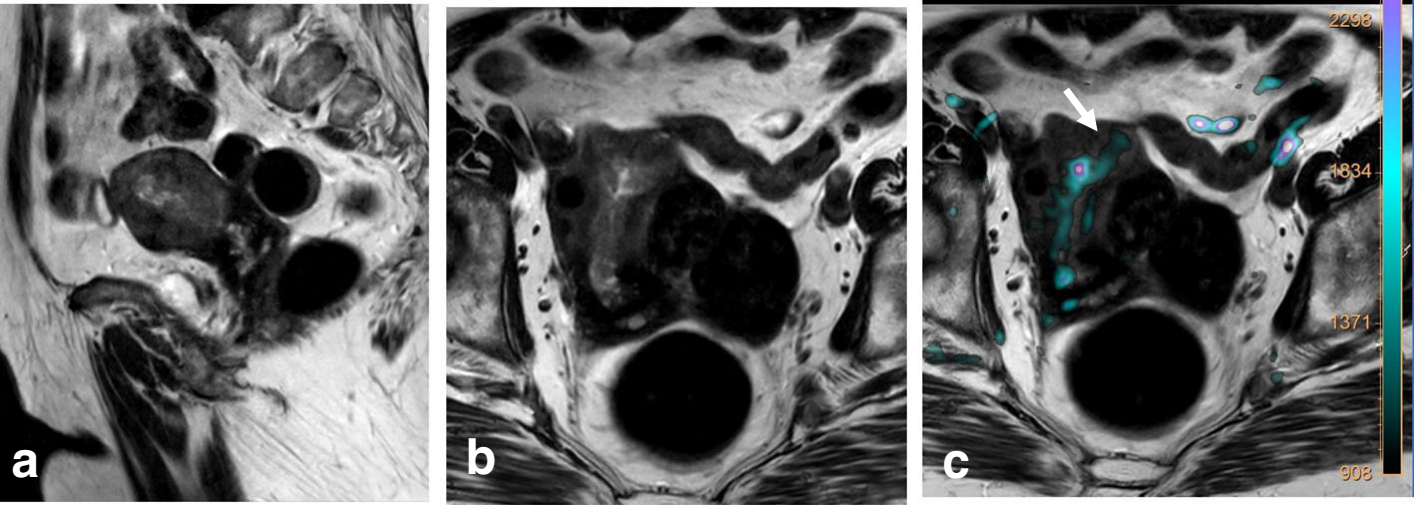
FIGO IB tumor exhibiting left cornual extension. **a** Axial oblique T2WI. **b** Coronal T2WI. **c** Fused T2WI-DW images show endometrial tumor (arrow), which was difficult to delineate on individual images

**Fig. 6:**
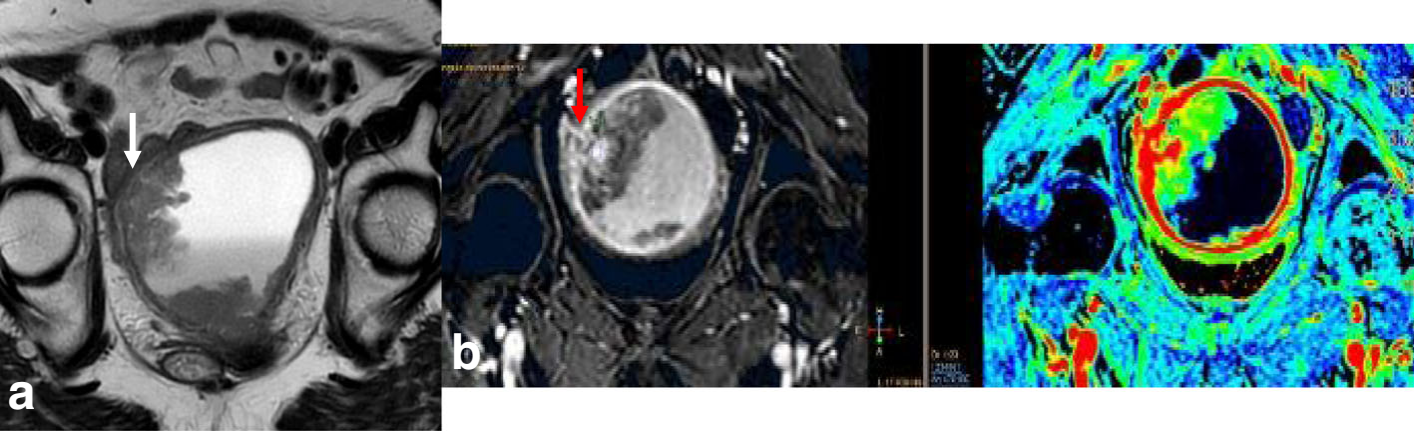
Endometrial cancer with endometrial cavity distension. **a** Axial oblique T2WI. **b** DCE and parametric map. Disruption of the endometrial–myometrial zone (arrows) indicates myometrial invasion

The following possible pitfalls can result in underestimation or overestimation of myometrial invasion (Table 5): no detection of isointense tumors on T2WI; poor endometrial–myometrial interface contrast due to the presence of fibroids or adenomyosis (Fig. 4); thin myometrium in cornual regions where myometrial thickness is difficult to measure, especially in older women who have an atrophic uterus and a less-discernible junctional zone (Fig. 5); and endometrial thinning due to either age or endometrial cavity distension (Fig. 6). In all of these scenarios, DWI and DCE imaging can help to detect and delineate tumors and to assess myometrial invasion (Table 5). On T2WI, uterine serosa invasion appears as an area of intermediate-to-high SI disrupting the normal smooth contour of the outer myometrium. On DCE images, loss of the normal rim of enhancement of the outer myometrium indicates serosal involvement [4, 8].

**Table 5:** MRI in endometrial cancer staging. Pitfalls and pearls

Staging (FIGO)	Pitfall	Pearl
1. STAGE IA/IB: detection and myometrial invasion • Small or isointense tumors • Poor visualization of endometrium and/or poor tumor-to-myometrium interface: *-* Presence of leiomyomas/adenomyosis - Thin myometrium: postmenopause, cornual regions, or secondary to a compressive large endometrial mass	No detection Underestimation or overestimation of myometrial invasion depth	*-* DCE and DWI improve detection of small and isointense tumors *-* DWI improves tumor detection and delineation *-* In DCE imaging, the presence of a contiguous band of subendometrial enhancement excludes myometrial invasion
2. STAGE II: cervical invasion Tumor protruding or distending cervical os	Misdiagnosis of cervical invasion	- Cervical stroma disruption is necessary for diagnosis of cervical stromal invasion - DWI and DCE improve tumor delineation
3. STAGE IIIA • Coexistent ovarian and endometrial tumor	Misinterpreting stage IIIA as synchronous cancer and vice versa	- Synchronous ovarian and endometrial cancer *•* Uterus: early-stage endometrial cancer with minimal or no myometrial invasion • Ovary: unilateral large mass in the background of endometriosis or borderline tumor - Ovarian metastasis (IIIA) • Uterus: deep myometrial invasion and/or tubal invasion • Ovary: smaller mass, bilateral ovarian involvement
4. STAGE IV Tumor invades bladder/bowel mucosa	The presence of bladder mucosal edema (bullous edema) is not indicative of mucosal invasion	- Change the direction of phase and frequency - A preserved fat plane between the tumor and bladder or rectum excludes stage IVA - DWI (DWI+T2WI) and DCE help in tumor delineation

#### Cervical invasion (stage II)

**Fig. 7:**
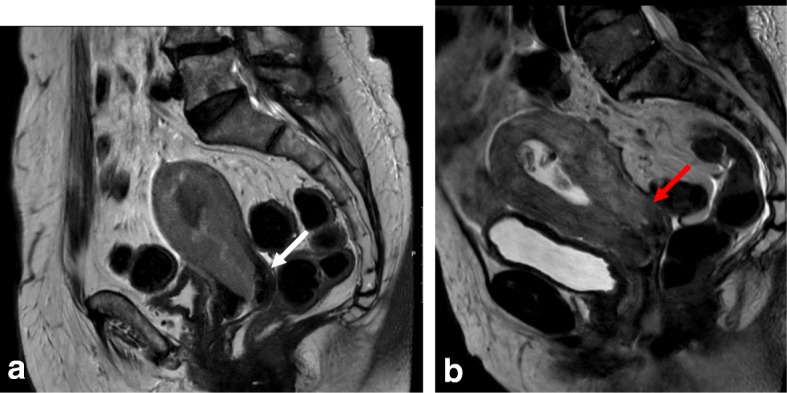
Endometrial cancers visualized by sagittal T2WI. **a** No cervical invasion; cervical stromal is preserved (white arrow). **b** Direct cervical invasion; cervical stroma is invaded (red arrow)

On T2WI, cervical stroma invasion is seen as a disruption of the normal low SI of the cervical stroma by the intermediate SI of the tumor. It can be difficult to ascertain by MRI whether the tumor protrudes only into the cervical canal or invades the cervical stroma. Even if a tumor distends the uterine and cervical cavity, invasion is only diagnosed if the cervical stroma is disrupted. Notably, direct cervical stroma invasion may occur without endocervical mucosa invasion in cases of adjacent myometrial invasion (Fig. 7). In DWI, cervical stroma invasion is suggested by the presence of high SI on high *b* values and lower SI on ADC maps disrupting the cervical stroma. On DCE imaging, cervical stroma invasion is defined by an interruption of the normal enhancement of the cervical stroma [4, 8, 64].

#### Adnexal or ovarian metastases (stage IIIA)

**Fig. 8:**
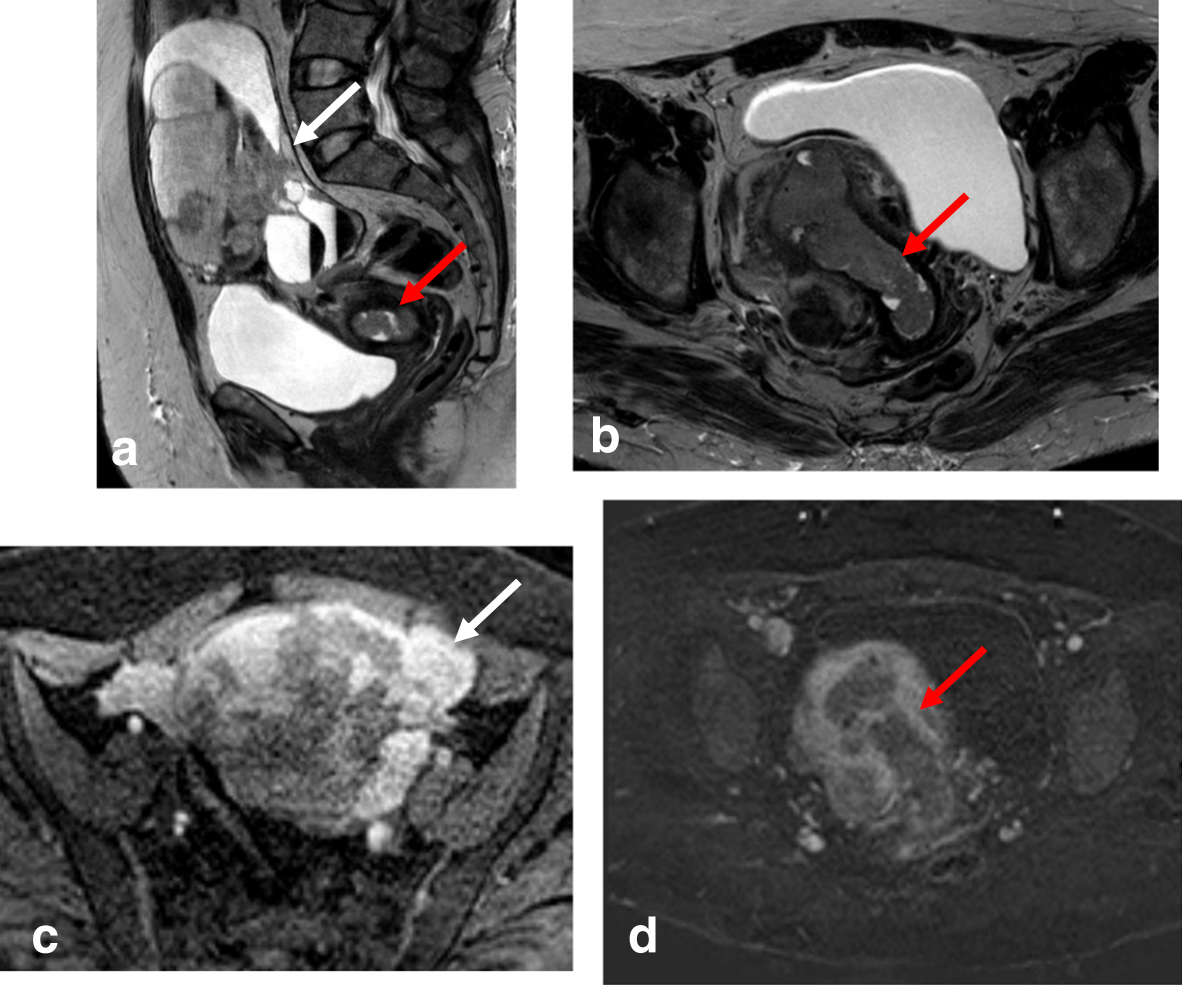
Synchronous endometrial and ovarian cancer. Visualization by sagittal T2WI (**a**) and axial oblique T2WI (**b**) reveals a large multicystic ovarian mass (white arrow) and endometrial cancer (red arrow). **c**, **d** DCE imaging shows heterogeneous uptake in the ovarian mass (white arrow) and hypoenhancement in the endometrial mass (red arrow)

Tumors showing direct spread to the adnexa or ovarian metastases are considered stage IIIA disease. In 5–8% of cases, synchronous primary ovarian carcinoma coexists with endometrial cancer [65]. Some features suggest ovarian metastasis, including bilateral ovarian involvement, morphological similarity between ovarian and uterine masses, and a larger uterine mass compared to the ovarian mass. Other features are more suggestive of primary synchronous tumors, such as the presence of a large unilateral ovarian mass, evidence of precursor ovarian lesions (e.g., endometriosis), and a low-grade uterine mass without deep myometrial invasion (Table 5; Fig. 8) [66, 67].

#### Vaginal metastasis (stage IIIB)

**Fig. 9:**
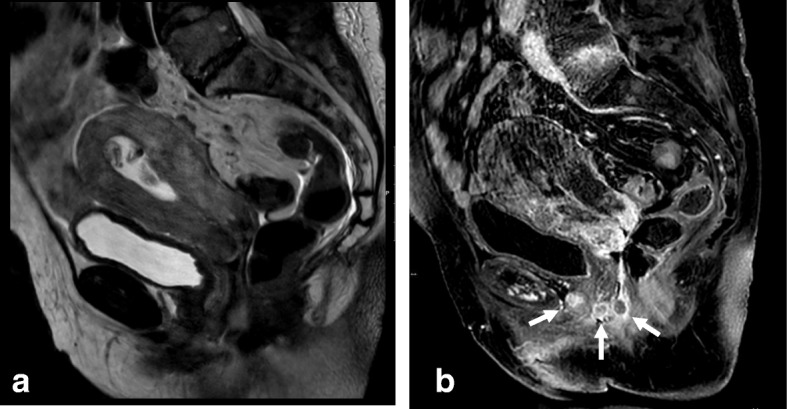
Small cell neuroendocrine tumor with myometrium and cervical invasion visualized by sagittal T2WI (**a**) and sagittal DCE (**b**). Vaginal and pubic metastasis are much better delineated in the DCE sequence (arrows)

Tumors that involve the vagina by either direct invasion or metastatic spread (“drop metastases”) are classified as stage IIIB tumors. DWI and DCE imaging are particularly helpful for detecting small cervical and vaginal implants (Fig. 9).

#### Bladder and bowel mucosa involvement (stage IVA)

Rectum and bladder wall invasion are best evaluated in the sagittal plane. Preservation of the fat plane between the tumor and bladder or rectum excludes stage IVA disease. Proper patient preparation is important since this anatomic plane may be difficult to delineate in patients with a full extended bladder. The presence of bladder mucosal edema (bullous edema) does not indicate mucosal invasion [2, 4, 8, 19].

#### Distant metastases (stage IVB)

Malignant ascites and peritoneal implants are more commonly found in cases of type II endometrial tumors. DWI can help detect small serosal/peritoneal deposits that may easily be missed on T2WI.

## Recurrence of endometrial cancer

In the follow-up of high-risk patients, CT is routinely used to identify recurrent disease within the lungs or LNs. However, the vaginal vault can be difficult to assess with CT, and MRI provides improved soft-tissue resolution. DWI and DCE imaging are particularly useful for distinguishing between postradiotherapy soft-tissue thickening and inflammation or recurrent disease [6, 20]. ^18^FDG PET-CT can be used to evaluate patients with clinically suspected recurrent disease that is not detected by conventional imaging [32].

## Mimickers of endometrial cancer on MRI

### Benign mimickers

**Fig. 10:**
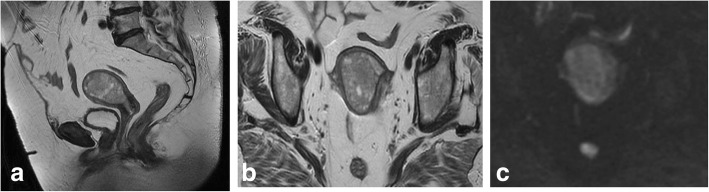
Endometrial hyperplasia visualized by sagittal T2WI (**a**) and axial oblique T2WI (**b**). Both images show intracavitary endometrial proliferation that is hyperintense relative to the myometrium. In panel **c**, there is no diffusion restriction on *b* = 1000

Differentiation between EC versus benign conditions, such as endometrial hyperplasia or polyps, requires careful inspection of T2WI, DWI, and DCE images (Fig. 10). The ADC values are significantly lower for EC compared to endometrial polyps and normal endometrium. Occasionally, a submucosal leiomyoma may be mistaken for focal thickening of the endometrium and thus considered an endometrial carcinoma—especially on TVUS. In this scenario, the low SI of leiomyomas on T2WI allows differentiation between these entities.

### Malignant mimickers

**Fig. 11:**
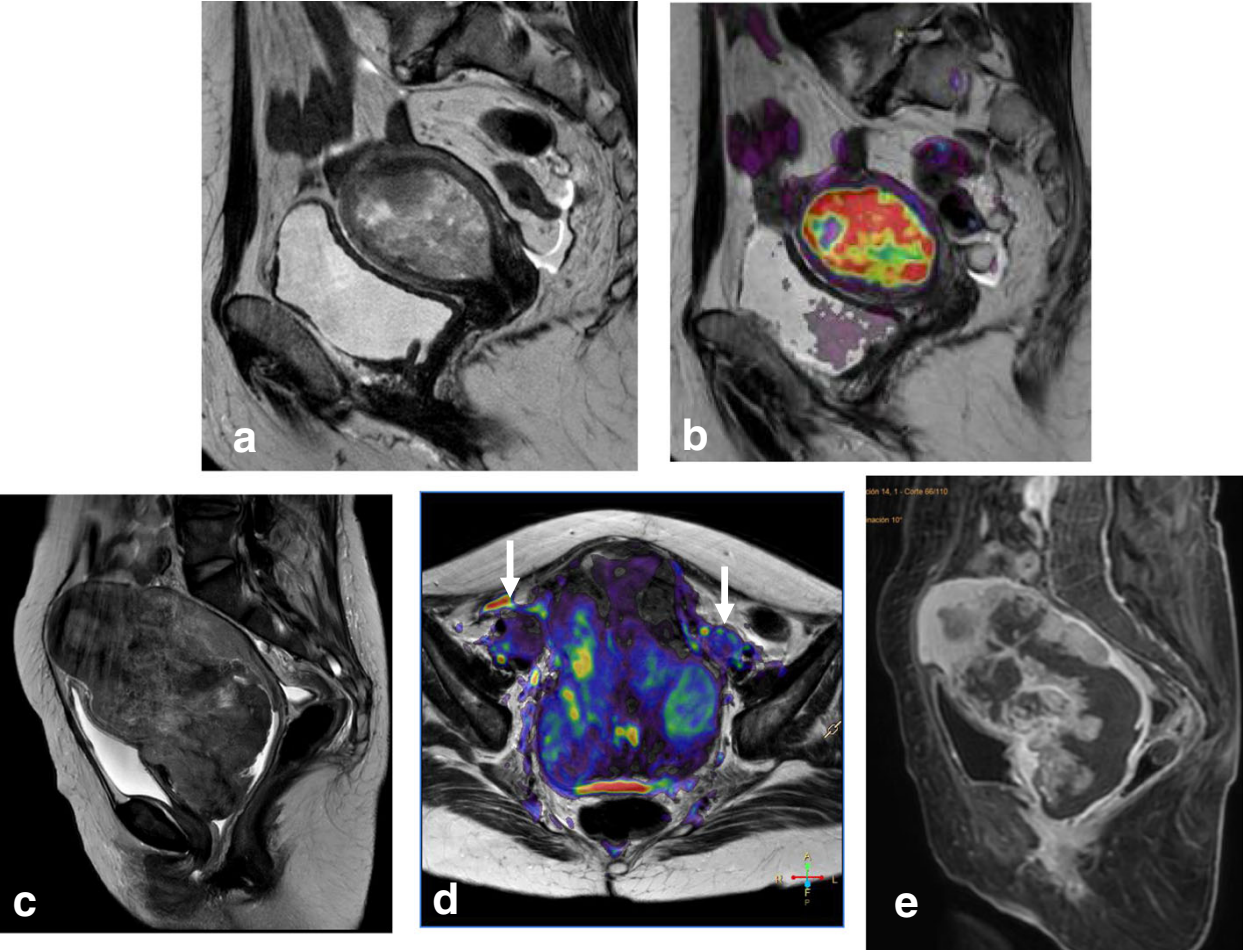
Endometrial-based tumor visualized with sagittal T2WI (**a**) and sagittal T2WI+DWI (**b**) was found to be a carcinosarcoma mimicking endometrial carcinoma. **c**–**e** In another patient, an endometrial stromal sarcoma mimicking cervical cancer is visualized by sagittal T2WI (**c**), axial T2WI+DWI (**d**), and DCE (**e**), revealing a cervical-centered mass with parametrial invasion and adenopathies (arrows in **d**)

Malignant mimickers include other endometrial-based tumors, such as endometrial stromal sarcoma and undifferentiated endometrial sarcoma or adenosarcoma. A correct diagnosis is made by endometrial biopsy or curettage and histopathological analysis (Fig. 11).

## MRI pitfalls in cervical cancer

The size, location, and extent of cervical tumors can be best evaluated using high-resolution, non-FS T2WI and DWI [10]. On T2WI, the tumor shows an intermediate-to-high SI compared to the uterine smooth muscle or myometrium. On DWI, the tumor exhibits an increased SI on high-*b*-value images and a corresponding low signal on ADC maps. On DCE images, small tumors exhibit increased early homogeneous hyper-enhancement relative to the adjacent normal cervix, whereas larger tumors often show heterogeneous enhancement secondary to necrosis [10].

### Staging pitfalls

#### Early tumors (stages IA–IB1)

MRI cannot delineate microinvasive tumors and thus plays no role in the evaluation of stage IA lesions. Recent studies report that MRI has a sensitivity of 90% and specificity of 98% for the staging of early IB1 tumors. The addition of DWI and DCE imaging enables the detection of tumors smaller than 1 cm (Table 6) [2, 32, 68].

**Table 6:** MRI in cervical cancer staging. Pitfalls and pearls

Staging (FIGO)	Pitfall	Pearl
1. STAGE IA, IB1 (< 2 cm) • Very small (< 1 cm) tumors • Isointense tumors in young women	No detection	• DWI and DCE improve detection and delineation of small tumors
2. STAGE IB3 • Cervical edema and/or inflammation secondary to a recent biopsy or to cervical/vaginal compression by a large tumor (> 4 cm)^a^	Overstaging IB3 as stage IIA in large and exophytic tumors Overstaging as FIGO IIB tumor (parametrial invasion)	• Use vaginal gel to distend vaginal walls • DWI and DCE improve the accuracy of T2WI for the evaluation of parametrial invasion Ancillary findings for parametrial invasion: • Irregular interface between tumor and parametrium • Asymmetric tumoral bulge • Vascular encasement
3. Stage IIB • Diffuse T2 signal inhomogeneity of the cervical rim due to complete tumoral invasion, without an evident parametrial mass	Understaging IIB as IB2–IB3 tumors	• Full-thickness cervical stromal replacement by cancerous tissue may be the only feature associated with parametrial invasion • The cervical rim must be thick (> 3 mm) and homogeneous on T2WI to exclude parametrial invasion
4. STAGE III • IIIB • IIIC	Misinterpreting a benign hydronephrosis as malignant ureteral infiltration Misinterpreting benign adenopathies as malignant lymphatic spread Misinterpreting malignant adenopathies as other pelvic masses (ovaries …)	• Review clinical data and symptoms, and use other techniques (i.e., ultrasound, CT urography, or large-FOV MRI). • Review clinical data and symptoms and perform node aspiration or biopsy whenever possible • Knowledge of pelvic fascia, peritoneal-extraperitoneal spaces, and other pelvic structures is critical
5. STAGE IV Same as in endometrial cancer (see Table 5)		

*FOV* field of view

^a^This pitfall can occur with any cervical mass (especially large masses) after biopsy because of peritumoral edema, or due to a false-positive estimation of vaginal invasion when the vaginal fornix is stretched by a bulky exophytic cervical tumor

#### Stage IB tumors

Stage IB tumors are now subdivided into three categories based on the lesion’s maximum diameter: stage IB1, diameter of < 2 cm and stromal invasion depth of ≥ 5 mm; stage IB2, diameter of ≥ 2 cm and < 4 cm; and stage IB3, diameter of ≥ 4 cm (Table 2) [26]. Acquisition of DWI sequences in the same plane as T2WI allows direct correlation, which helps delimit the tumor and improves the detection of small infiltrative tumors (IB1, IB2). On DCE images, small tumors may show earlier enhancement than adjacent stroma [10, 64–66].

**Fig. 12:**
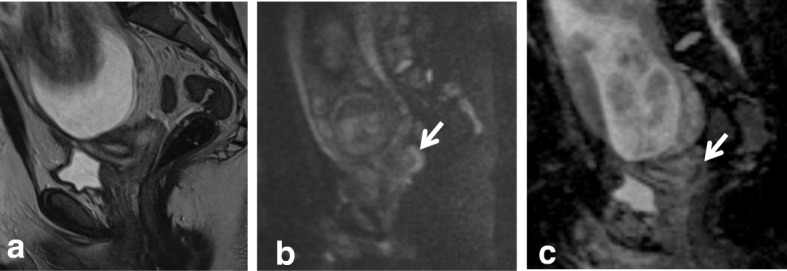
Pregnant woman with biopsy-confirmed cervical cancer that is not detectable on T2WI (**a**), but is delineated (white arrows) on DWI images (**b**, **c**)

In young patients, hormonal changes during the menstrual cycle may result in a higher SI of the cervix on T2WI, similar to that of the myometrium. DWI and DCE improve the delineation and tumor-to-cervical stromal contrast of small isointense tumors that are difficult to see on T2WI (Fig. 12) [10, 11, 34, 35].

#### Vaginal invasion (stage IIA)

Invasion of the upper two-thirds of the vagina corresponds to FIGO stage IIA. Stage IIA tumors are divided into stage IIA1 (tumors ≤ 4 cm) and stage IIA2 (tumors > 4 cm) based on prognostic differences between these groups. MRI shows high accuracy (86–93%) for assessment of vaginal invasion. Vaginal infiltration is identified by a hyperintense lesion disrupting the hypointense wall on T2WI and early contrast uptake after contrast administration (Table 6) [10, 11, 34].

**Fig. 13:**
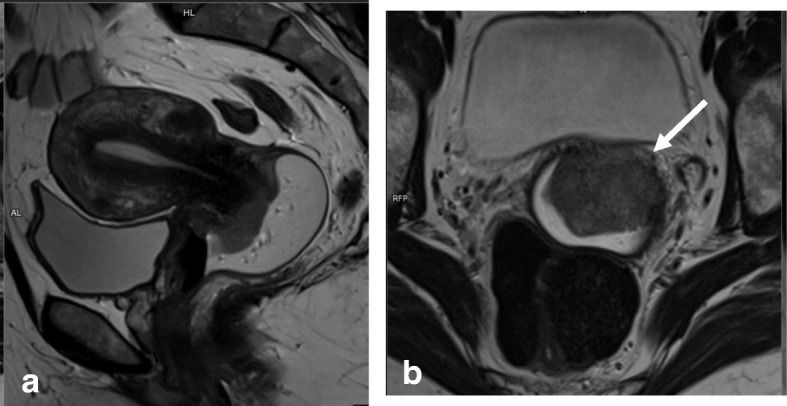
**a**, **b** Sagittal and axial oblique T2WI with vaginal gel, revealing a cervical carcinoma with vaginal infiltration, seen as disruption of the hypointense anterior wall (arrow)

A false-positive estimation of vaginal invasion usually occurs when the vaginal fornix is stretched by a bulky exophytic cervical tumor (FIGO IB3). Intravaginal gel can be used to improve vaginal fornix evaluation by separating the vaginal walls, improving the identification of minimal fornix involvement (Table 6; Fig. 13) [43, 69].

#### Parametrial invasion (stage IIB)

**Fig. 14:**
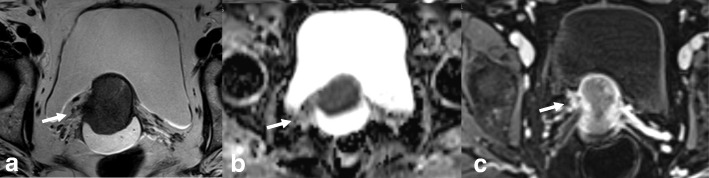
Tumor visualization with axial oblique T2WI (**a**), DWI-ADC map (**b**), and DCE imaging (**c**) reveals loss of the hypointense cervical rim on the right lateral aspect of the cervix, with an irregular interface between the tumor and the parametrium (arrow). ADC map shows diffusion restriction, and DCE imaging reveals enhancement of the right lateral wall indicating early parametrial invasion (arrows)

Tumor extension to the parametrial fat implies disruption of the hypointense cervical stromal rim, with extension of nodular or spiculated soft tissue into the adjacent parametrium (Fig. 14). MRI shows 88–97% accuracy in appropriately evaluating parametrial extension. High-resolution T2WI in the axial oblique plane is essential for assessing the disruption or preservation of the hypointense stromal rim. Preservation of a stromal rim thickness of > 3 mm excludes parametrial involvement (specificity, 96–99%; NPV, 94–100%) [2, 10, 70, 71]. Notably, in patients exhibiting full-thickness cervical stromal replacement by cancerous tissue, diffuse T2 signal inhomogeneity of the cervical rim may be the only feature associated with parametrial invasion. Therefore, accurate exclusion of parametrial involvement requires that the cervical rim be thick (> 3 mm) as well as homogeneous [71].

**Fig. 15:**
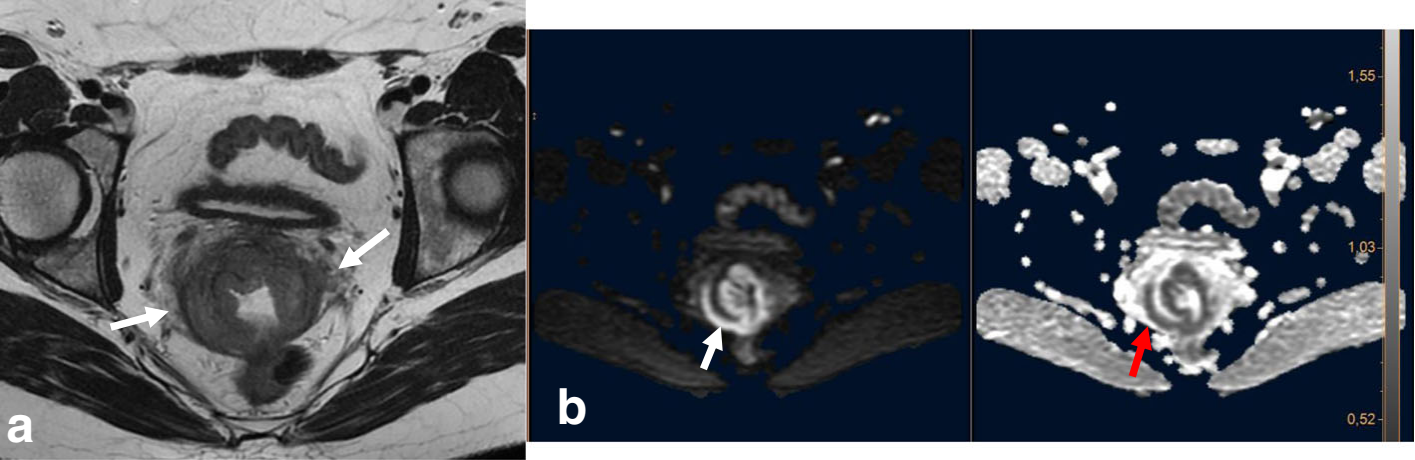
FIGO IB2 cervical cancer, with post-biopsy cervical edema. Biopsy was performed 2 days before MRI. **a** Axial oblique T2WI shows cervical cancer with apparent parametrial invasion (arrows). **b** DWI (*b* = 1000/ADC map) reveals a delineated tumor (arrows), without parametrial invasion

Signs of parametrial invasion can also include ancillary findings—such as the presence of an irregular interface between the tumor and parametrium, an asymmetric tumoral bulge, and a peri-uterine vessel encasement in low cervical tumors (Table 6) [2, 10, 70]. Cervical edema and/or inflammation secondary to a recent biopsy, and cervical compression by a large diffuse infiltrating tumor, can be misinterpreted as parametrial invasion, potentially leading to the misdiagnosis of a FIGO IB3 tumor as FIGO IIB*.* DWI and DCE imaging can help avoid the overestimation of inflammation and improve the accuracy of T2WI for evaluating parametrial invasion (Fig. 15) [2, 10, 68].

#### Stage III

**Fig. 16:**
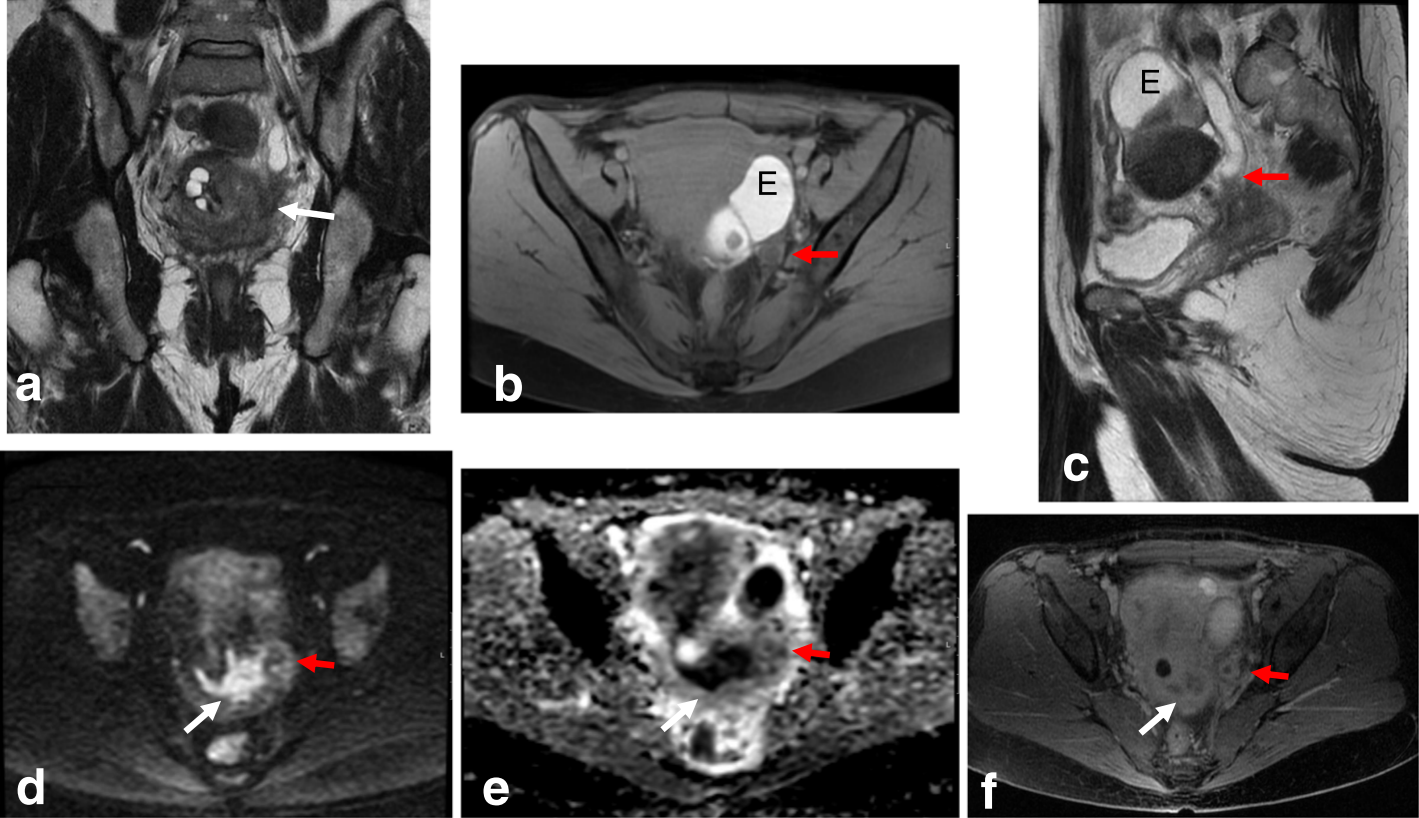
MRI reveals FIGO IIIB cervical cancer in a 38-year-old woman with adenomyosis and endometriosis in the left ovary. Visualization by coronal T2WI (**a**), axial FS T1WI (**b**), and sagittal T2WI (**c**) reveals a mass extending to the left parametrium and involving the left ureter (red arrows) below the endometrioma (E), with a subsequent hydronephrosis. **d**, **e** DWI (*b* = 1000/ADC map) reveals diffusion restriction of the cervical mass, with extension to the left ureter. **f** DCE sequence shows enhancement of the cervical mass and ureter wall

Stage III is now subdivided into three categories. Stage IIIA disease is characterized by involvement of the lower third of the vagina, where the normal hypointense T2W signal of the lower third of the vaginal vault is disrupted by a hyperintense tumor. Stage IIIB tumors exhibit extension to the pelvic wall and/or hydronephrosis or nonfunctioning kidney. Other causes of hydronephrosis (i.e., endometriosis or lithiasis) should be excluded to avoid misdiagnosis of stage IIB disease (Table 6; Fig. 16) [26]. Stage IIIC disease is characterized by the presence of positive adenopathies (FIGO 2018). Stage IIC is further subdivided into IIIC1 (adenopathies located in the pelvis) or IIIC2 (paraaortic adenopathies) (Table 2). When large adenopathies are found, inflammatory and infectious diseases (i.e., tuberculosis and HIV) should be excluded. An accurate clinical history, including all clinical and analytical data, together with fine-needle aspiration or biopsy of the LNs, are critical for establishing a correct diagnosis (Table 6) [26].

#### Bladder and rectal infiltration (stage IVA)

Tumors extending into the mucosa of the bladder or rectum are classified as stage IVA. MRI is an accurate technique for evaluating bladder or rectum involvement, with a sensitivity of 71–100% and specificity of 88–91% [2, 10, 11]. The bladder and rectum are evaluated by cystoscopy and sigmoidoscopy only when the patient is clinically symptomatic. Cystoscopy is also recommended in cases showing a barrel-shaped endocervical growth or where the growth extends to the anterior vaginal wall [26].

**Fig. 17:**
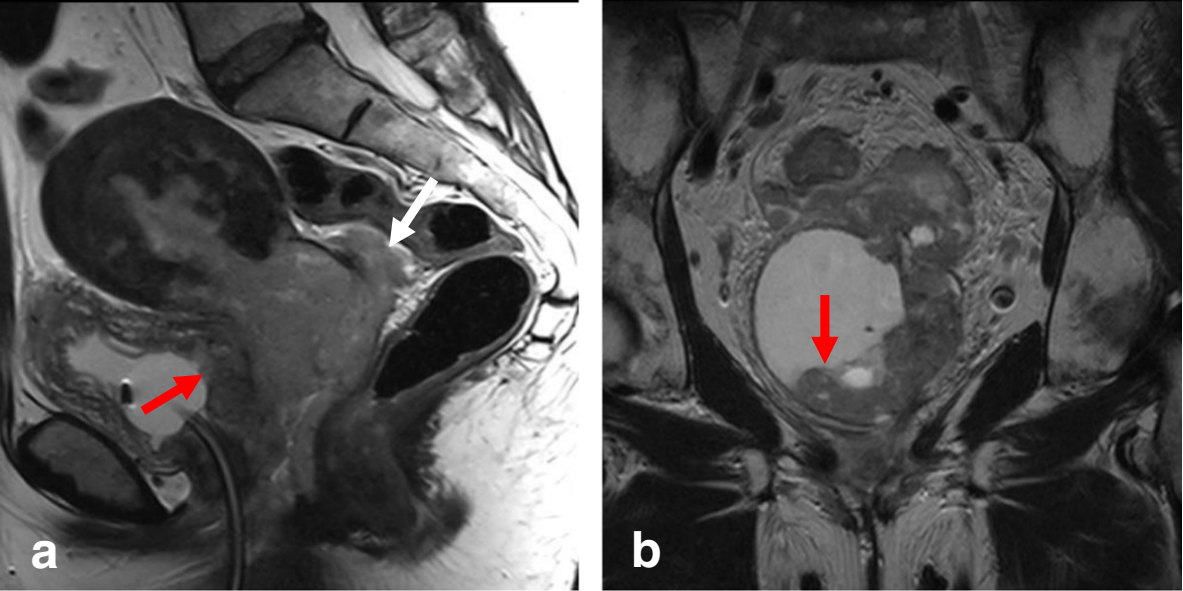
FIGO Stage IVA cervical cancer with bladder invasion. Visualization by sagittal T2WI (**a**) and coronal T2WI (**b**) reveals cervical cancer invading the bladder mucosa (red arrow) and rectouterine space (white arrow)

**Fig. 18:**
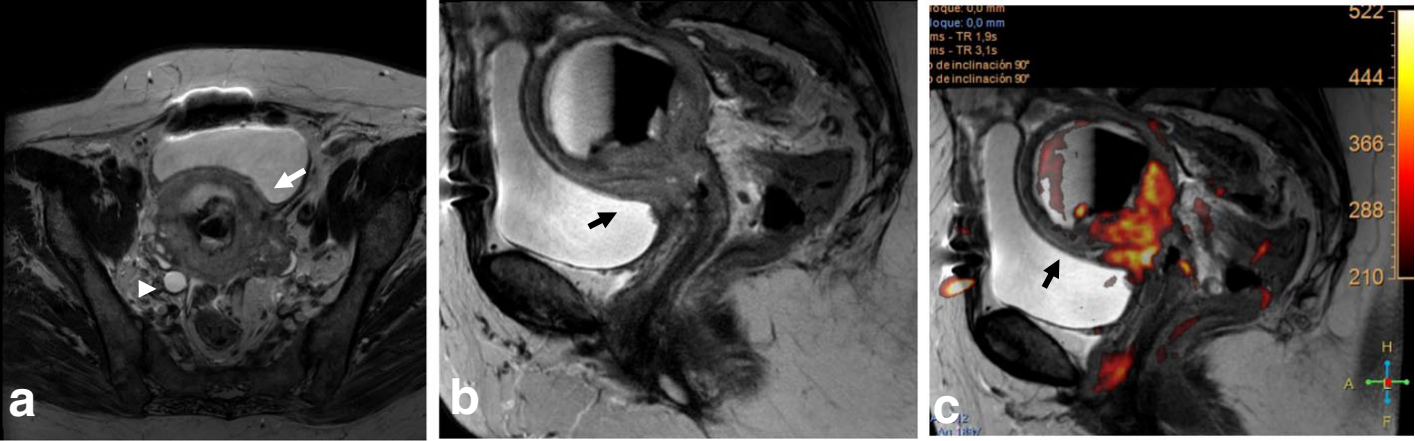
Cervical cancer. **a** Axial oblique T2WI shows a cervical mass (arrow), with hematometra and ureteral invasion (arrowhead) (IIIB). **b** Sagittal T2WI shows loss of the hypointense rim of the posterior bladder wall suggesting invasion (arrows). **c** T2WI+DWI reveals no invasion of the bladder wall (arrow)

Bladder or rectal invasion is suspected in cases showing obliteration of the fatty plane between the cervix and adjacent organs. Tumor presence in the lumen of the bladder or rectum is an unequivocal sign of infiltration and should be confirmed by biopsy and histologic analysis (Fig. 17) [24, 39]. A vesico-cervical fistula is also a sign of advanced disease. DWI and DCE allow better detection of fistula tracts [24, 39, 72]. Bullous edema can lead to false estimation of posterior wall bladder invasion (Fig. 18)*.* DWI and DCE imaging can also help to avoid misinterpreting bullous edema as invasion [2, 11, 70].

#### Distant metastasis (stage IVB)

Stage IVB disease is characterized by the involvement of the paraaortic or inguinal lymph nodes, liver, lung, and/or bones [10].

## Recurrence of cervical cancer

**Fig. 19:**
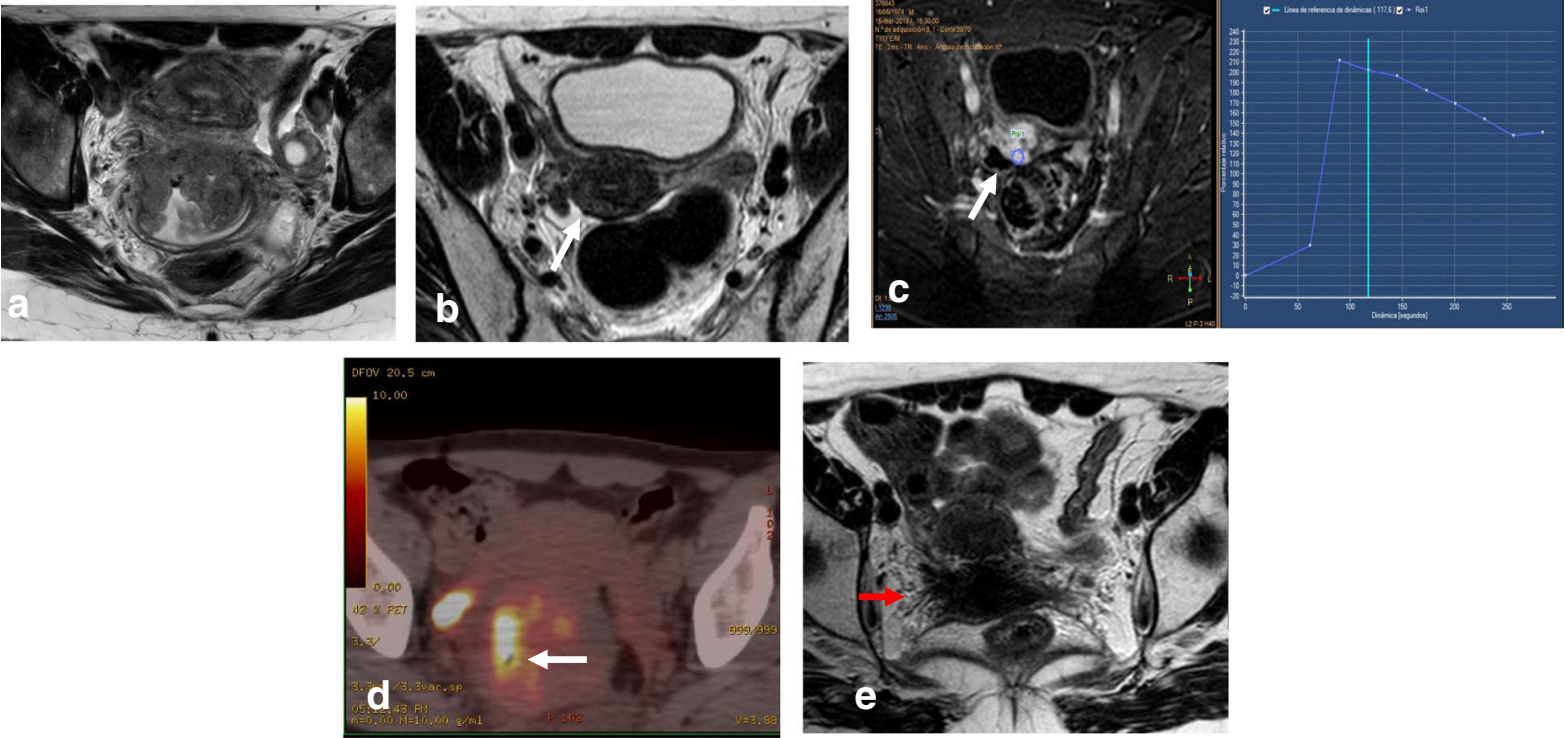
Cervical cancer treated with chemotherapy and radiotherapy. **a** Axial oblique T2WI at diagnosis shows a cervical mass. **b**, **c**, **d** A post-treatment residual mass (arrows) is detected on the right posterior aspect of the cervix by axial oblique T2WI (**b**), DCE and a type 3 time-intensity curve (**c**) and PET/CT (**d**). **e** Axial oblique T2WI at 6 months after treatment establishes cervical and right parametrium fibrosis (red arrow) and shows no residual mass

MRI is the best imaging technique for patient follow-up and for detecting local recurrence after treatment [2, 11]. On T2WI, recurrent disease manifests as a mass with intermediate-to-high SI [2, 11]. In patients treated with radiotherapy, it is essential to differentiate between post-radiation changes and local recurrence. Administration of intravenous contrast improves detection of recurrence, as recurrence shows early enhancement (45–90 s) and restriction on DWI sequences, whereas fibrosis exhibits no significant enhancement or enhances in late phases. Both tumor recurrence and inflammatory changes may show hyperintensity at a high *b* value, but only recurrent tumors will have lower SI on the ADC map [73]. These patients may require evaluation by image-guided biopsy or ^18^FDG PET-CT (Fig. 19).

## Mimickers of cervical cancer on MRI

Both benign and malignant cervical diseases can mimic CC. MRI is a useful technique for distinguishing between these entities. Invasion of the cervical stroma, contrast enhancement, and low values on ADC maps are highly suggestive of malignancy. However, biopsy is sometimes necessary.

### Benign mimickers

**Fig. 20:**
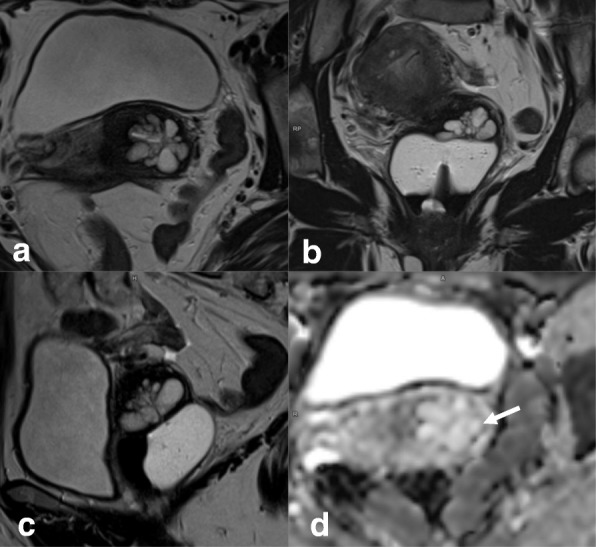
Tunnel clusters visualized by axial oblique T2WI (**a**), coronal T2WI (**b**), sagittal T2WI (**c**), and DWI-ADC map (**d**). Clustered cystic spaces have a round or oval appearance, an absence of invasion of the deep cervical stroma, and no diffusion restriction (arrow in **d**)

Nabothian cysts are generally incidental findings. They are hyperintense on T2WI and may have variable T1 signals. Nabothian cysts typically have no solid enhancing components. The tunnel cluster is a type of Nabothian cyst that shows complex cystic dilatation of the endocervical glands (Fig. 20). Identifying solid parts is the key to differentiating a tunnel cluster from neoplasms with a cystic component, such as adenoma malignum [74].

Cervical polyps are common in women during the fifth decade of life and are the most frequent cause of intermenstrual bleeding in perimenopausal women. The classical cervical polyp arises from the cervical canal and exhibits a predominantly glandular structure with a fibrous core [75].

### Malignant mimickers

Adenoma malignum (also termed minimal deviation adenocarcinoma) is a subtype of mucinous adenocarcinoma of the cervix, which accounts for 3% of all cervical adenocarcinomas. It exhibits early dissemination and poor prognosis. Adenoma malignum has been associated with Peutz-Jeghers syndrome, ovarian mucinous tumor, and ovarian sex cord tumor. The most common symptom is a watery vaginal discharge [75, 76].

**Fig. 21:**
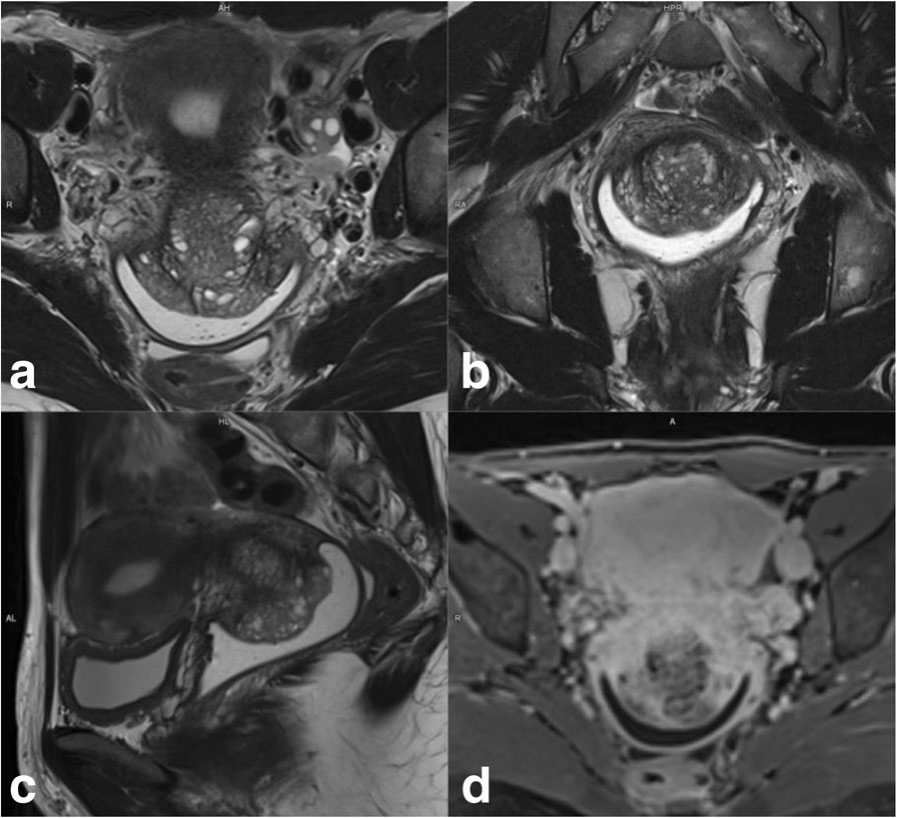
Adenoma malignum visualized by axial oblique T2WI (**a**), coronal T2WI (**b**), sagittal T2WI (**c**), and DCE (**d**). Multicystic lesions extend from the endocervical glands to the deep cervical stroma with solid components, forming a mass (arrows)

On MRI, adenoma malignum appears as a multicystic lesion with a solid component located in the endocervical glands, which extends to the deep stroma, showing a very high T2 signal and slight hyperintensity on T1WI. The solid component may exhibit diffusion restriction on DWI and enhancement after contrast administration on DCE imaging (Fig. 21) [76].

**Fig. 22:**
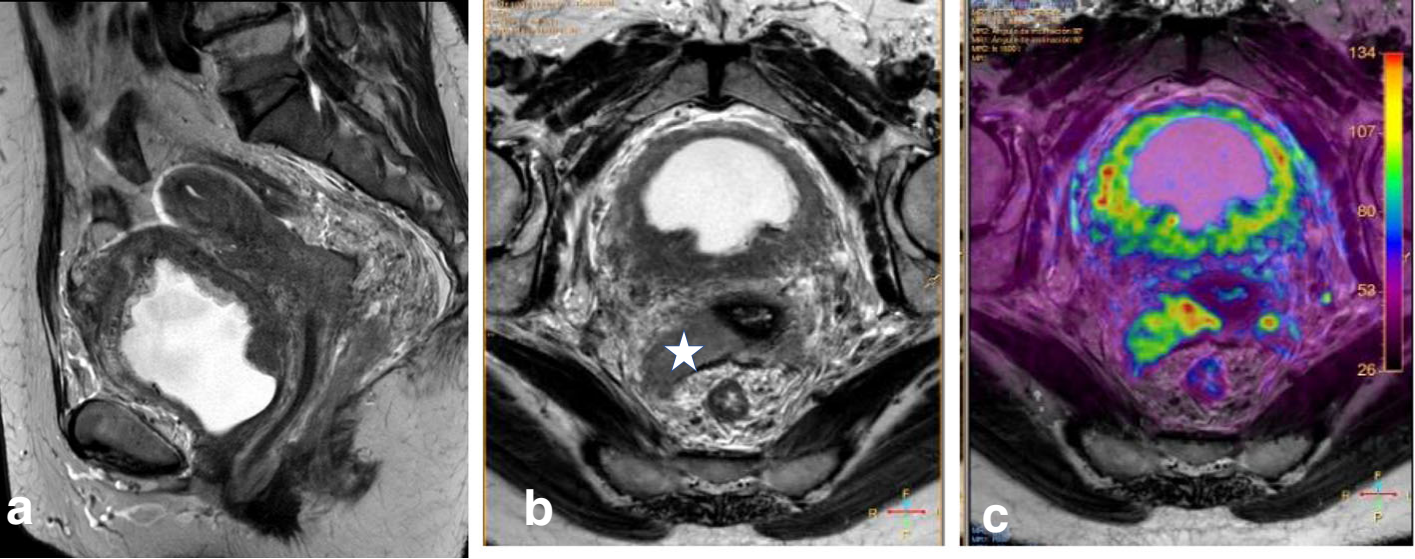
Transitional bladder cancer invading the cervix (star), visualized by sagittal T2WI (**a**), axial oblique T2WI (**b**), and T2WI+DWI (**c**)

Large pelvic tumors of the uterus, rectum, bladder, or unknown origin can also mimic CC stage IVA disease. Recto-sigmoidoscopy and/or cystoscopy may be necessary to determine the tumor origin or to exclude secondary bladder or rectal invasion by CC (Fig. 22).

### Mimickers of recurrence

**Fig. 23:**
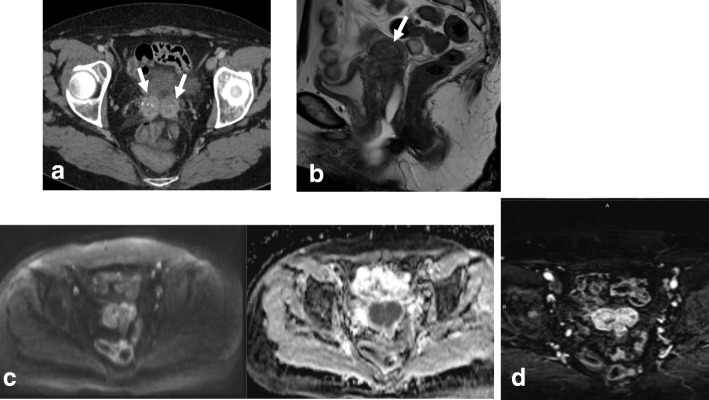
Vaginal vault mass after surgery for cervical carcinoma (white arrows) visualized by sagittal T2WI (**a**), axial oblique T2WI (**b**), DWI (*b* = 1000, ADC map) (**c**), and DCE (**d**). Imaging reveals diffusion restriction and early contrast uptake. Biopsy and histopathology indicated granulation tissue

Post-surgical inflammatory lesions—such as granulation tissue, abscesses, or cicatricial endometriosis—can simulate relapse. On DWI, they appear as masses with diffusion restriction, usually in the vaginal recess or anastomotic area (secondary to the presence of high cellular and blood content). Clinical history, laboratory findings, and biopsy will help establish the correct diagnosis (Fig. 23).

### Cervical versus endometrial cancer

It is critically important to distinguish between cervical and endometrial carcinoma due to differences in staging, prognosis, and therapeutic approach. In a small number of patients with bulky uterine masses, conventional biopsy may not be sufficient to establish a cervical or endometrial origin. In such cases, immunohistochemistry may help but no single antibody is entirely specific for cervical or endometrial cancer. Some MRI features may discriminate between primary cervical and endometrial carcinomas, such as the presence of an endometrial mass in patients with EC [77]. Tumor epicenter determination with MRI reportedly shows high accuracy (up to 88%) for identification of tumor origin [78, 79].

Bourgioti et al. [80] described a MRI scoring system that discriminates between endometrial and cervical cancer based on seven features: tumor epicenter (uterine body or cervix), presence of tumor hypervascularity on early arterial DCE-MRI, full-depth cervical stromal invasion on T2WI images, mass within the endometrial cavity, distended endometrial cavity with secretions, deep (≥ 50%) myometrial invasion, and enhancing rim at the tumor periphery. Based on the possible likelihood ratio values assigned to each of the above features, this scoring system shows highly accurate tumor origin prediction, with sensitivity up to 96.6% and specificity up to 100%.

## Lymphatic dissemination in endometrial and cervical cancer

**Fig. 24:**
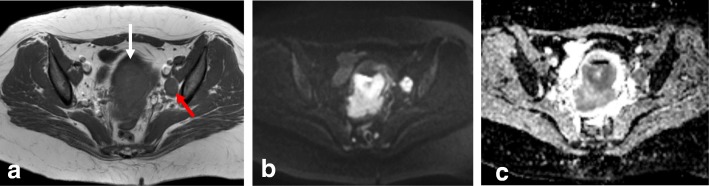
Axial oblique T2W (**a**) and DWI images (**b**, **c**) showing stage IIIC 1 endometrial cancer (white arrow) with metastatic left obturatory lymph node (red arrow)

For both endometrial and cervical cancer, assessing LN involvement plays an important role in tumor staging, treatment planning, and prognosis [5, 8, 10, 24, 26, 39]. Based on LN metastases, endometrial cancer is subdivided into stage IIIC1 (pelvic nodes) (Fig. 24) and stage IIIC2 (paraaortic nodes) (Table 1). In EC, risk factors for LN metastases include type II tumors, grade 3 tumors, LVSI, and deep myometrial invasion [4, 8]. LN metastasis distribution is influenced by EC tumor location. The middle and lower parts of the uterus drain into the parametrium and the paracervical and obturator nodes, while the upper part of the uterus drains into the common iliac and paraaortic LNs. Inguinal nodes are not part of the regional lymphatic drainage pathway for EC and, thus, if affected, represent stage IV disease. In CC, FIGO stage IIIC is also subdivided into IIIC1 (pelvic nodes) and IIIC2 (paraaortic nodes) (Table 2). Lymphatic spread of CC occurs along the obturator, external iliac, internal iliac, common iliac, and paraaortic nodes [26].

For discriminating between benign and malignant nodes, the most widely accepted criterion is nodal size (short axis > 1 cm) [34, 35]. MRI has moderate sensitivity (43%) and specificity (73%) for detection of metastatic lymph nodes. This is because MRI cannot discriminate between enlarged inflammatory lymph nodes and metastatic nodes and exhibits dissatisfactory diagnostic accuracy in cases of micrometastases [34, 81].

Malignant infiltration of LNs may also be indicated by morphologic criteria, such as a spherical shape and irregular contour, altered SI on T2WI, central necrotic areas, and nodal group formation. Central node necrosis has a PPV of 100% for the diagnosis of nodal metastases [34].

**Fig. 25:**
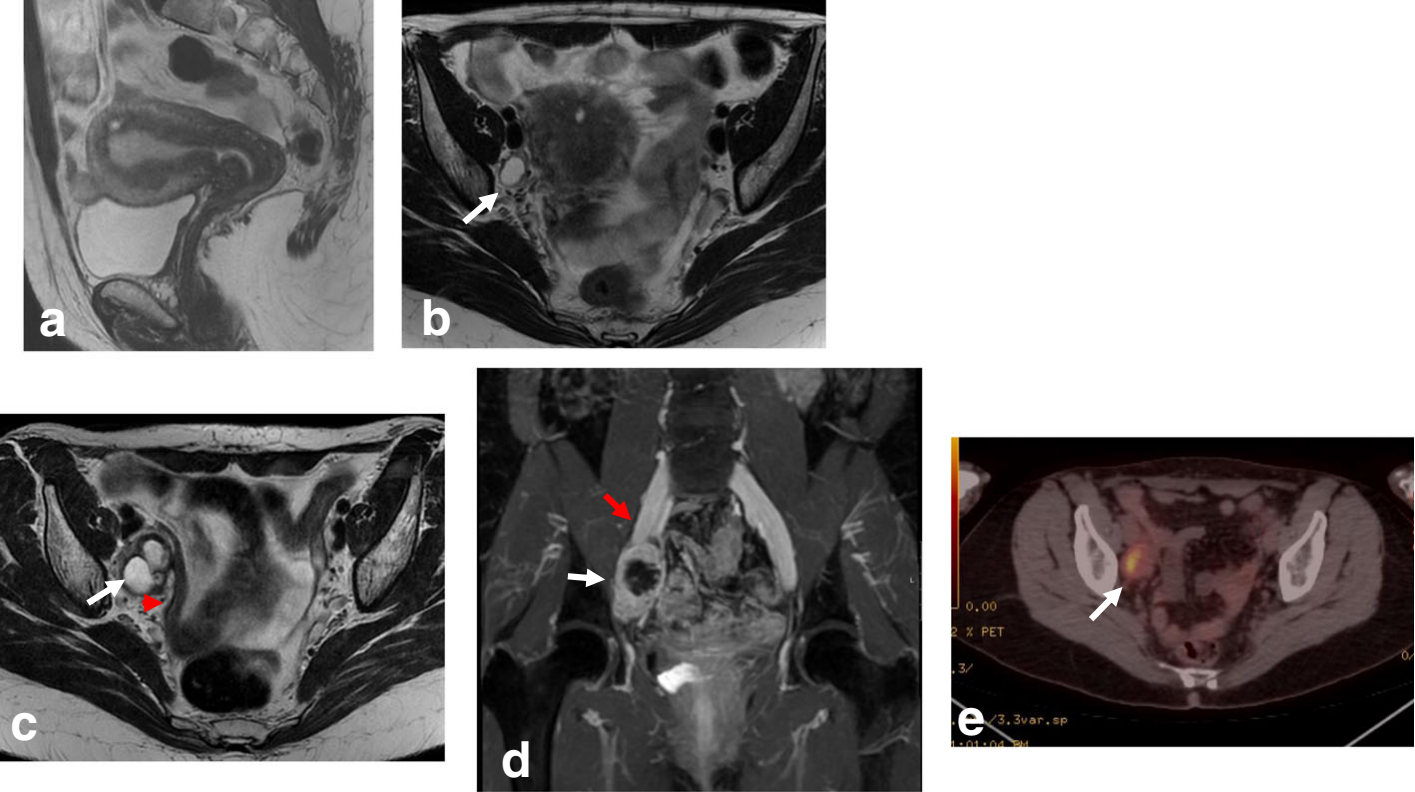
**a**, **b** MRI of a 48-year-old woman with known adenomyosis, a previous cesarean section, and FIGO IA1 cervical cancer diagnosed in an outpatient clinic, including sagittal T2WI (**a**) and axial oblique T2WI (**b**). No anomalies are visible in the cervix. The cystic image in the right obturator space (arrow in **b**) was interpreted as a right ovary with a small follicle. The patient underwent simple extrafascial hysterectomy with ovarian removal. Post-surgical histology confirmed FIGO stage IA1 (high-grade squamous cell carcinoma), without LVSI and with a clear surgical margin. **c**, **d** MRI was performed 6 months after surgery, including axial oblique T2WI (**c**) and coronal MIP-DCE sequence imaging (**d**). The right obturator cystic lesion increased in size and in contact with right iliac vessels (arrows). Images also show medial displacement of the right pelvic peritoneal fascia (red arrowhead) indicating an extraperitoneal origin of the cystic lesion. **e** ^18^F-FDG PET-CT shows avid enhancement of the cystic mass. Lymphadenectomy was performed. Histological analysis showed metastatic adenopathy of cervical cancer

Normal ovaries with follicles or small masses, neural or radicular tumors, and other pelvic masses can be misinterpreted as pelvic metastatic adenopathies. This pitfall can be avoided by careful evaluation of the pelvic structures; identification of peritoneal spaces, extraperitoneal spaces, and lymphatic channels; and analysis of clinical data (Table 6; Fig. 25) [34].

## Abbreviations

^18^F-FDG PET-CT: ^18^Fluorine-18 fluorodeoxyglucose PET-CT
ART: Abdominal radical trachelectomy
CC: Cervical cancer
CCRT: Concurrent chemoradiation
CT: Computed tomography
EC: Endometrial cancer
ESMO: European Society of Medical Oncology
ESUR: European Society of Urogenital Radiology
FIGO: International Federation of Gynecology and Obstetrics
FOV: Field of view
FS: Fat suppression
GRE: Gradient-echo-sequence
HPV: Human papillomavirus
LN: Lymph node
LNs: Lymph nodes
LVSI: Lymphovascular space invasion
NCCN: National Comprehensive Cancer Network
SI: Signal intensity
SLNB: Sentinel lymph node biopsy
T1WI: T1-weighted imaging
T2WI: T2-weighted imaging
TVUS: Transvaginal ultrasonography
VRT: Vaginal radical trachelectomy
